# Lancet2: Improved and accelerated somatic variant calling with joint multi-sample local assembly graphs

**DOI:** 10.1093/nargab/lqag036

**Published:** 2026-04-07

**Authors:** Rajeeva Lochan Musunuri, Bryan Zhu, Wayne E Clarke, William Hooper, Timothy Chu, Jennifer Shelton, André Corvelo, Dickson Chung, Shreya Sundar, Adam M Novak, Benedict Paten, Michael C Zody, Nicolas Robine, Giuseppe Narzisi

**Affiliations:** New York Genome Center, New York, NY, United States; New York Genome Center, New York, NY, United States; New York Genome Center, New York, NY, United States; Outlier Informatics Inc., Saskatoon, SK, Canada; New York Genome Center, New York, NY, United States; New York Genome Center, New York, NY, United States; New York Genome Center, New York, NY, United States; New York Genome Center, New York, NY, United States; UC Santa Cruz Genomics Institute, University of California, Santa Cruz, CA, United States; UC Santa Cruz Genomics Institute, University of California, Santa Cruz, CA, United States; UC Santa Cruz Genomics Institute, University of California, Santa Cruz, CA, United States; UC Santa Cruz Genomics Institute, University of California, Santa Cruz, CA, United States; New York Genome Center, New York, NY, United States; New York Genome Center, New York, NY, United States; New York Genome Center, New York, NY, United States

## Abstract

Here, we present Lancet2, an open-source somatic variant caller designed to improve detection of small variants in short-read sequencing data. Lancet2 introduces significant enhancements, including: (i) Improved variant discovery and genotyping through partial order multiple sequence alignment of assembled haplotype contigs, and re-alignment of sample reads to the best supporting allele. (ii) Optimized somatic variant scoring with explainable machine learning models, leading to better somatic filtering throughout the sensitivity scale. (iii) Integration with Sequence Tube Map for enhanced visualization of variants with aligned sample reads in graph space. When benchmarked against enhanced two-tech truth sets generated using high-coverage short-read (Illumina) and long-read (Oxford Nanopore) data from four well characterized matched tumor/normal cell lines, Lancet2 outperformed other industry-leading tools in variant calling performance, especially for InDels. In addition, significant runtime performance improvements were observed compared to Lancet1 (∼10× speedup and 50% less peak memory usage), and most other state-of-the-art somatic variant callers (at least 2× speedup with eight cores or more), making Lancet2 an ideal tool for accurate and efficient somatic variant calling.

## Introduction

Accurate and reliable detection of somatic Single Nucleotide Variants (SNVs) and insertions/deletions (InDels) is crucial for understanding the clonal structure and evolutionary dynamics of cancer. However, achieving comprehensive characterization of complex InDels remains a significant challenge for existing alignment-based somatic variant callers, largely due to reference bias [1]. Lancet addressed this issue by performing local assembly and jointly analyzing tumor and normal data with colored de Bruijn graphs, thereby reducing reference bias and improving sensitivity for InDels in the 30–250 bp “twilight zone” [2, 3].

Since its introduction, Lancet has become an integral part of the cancer genomics pipeline at the New York Genome Center [4] for liquid biopsy [5] and other efforts [6–8]. Its effectiveness has also been demonstrated in a variety of independent studies across multiple cancer types, including melanocytic naevi [9], polymorphous adenocarcinoma [10], pancreatic cancers [11, 12], colorectal cancer [13], hyalinizing trabecular tumors of the thyroid [14], and triple-negative breast cancer [15].

Despite its success and increasing adoption, several limitations remain in the original Lancet framework. These included relatively long runtimes, high memory usage, and the absence of an interactive visualization tool to examine somatic variants with read support in graph space. To address these shortcomings, we developed Lancet2—a redesigned and enhanced version of Lancet that offers improved performance, a new genotyping strategy, and advanced somatic variant scoring with custom glass-box boosting models using InterpretML [16–18]. The scope and recommended usage of Lancet2 are identical to that of Lancet1 [2] and is intended to be it’s successor/drop-in replacement. Like the original version [2], Lancet2 is meant to be used with tumor and matched normal short-read Illumina exome (or) whole-genome data for calling somatic SNVs and small InDels.

In this work, we compare Lancet2 against Lancet1 [2] and several industry-leading somatic variant callers, including Mutect2 [19], Strelka2 [20], DeepSomatic [21], and VarNet [22]. To ensure thorough benchmarking, we developed enhanced “two-tech” truth sets for four widely used cancer cell lines—HCC1187, HCC1143, COLO829, and HCC1395 [4, 23]—by integrating both high-coverage short-read Illumina and long-read Oxford Nanopore sequencing data. Our results demonstrate that Lancet2 not only improves variant calling accuracy, especially for InDels, but also significantly reduces runtime and memory usage while offering intuitive visualization tools to facilitate variant interpretation.

## Methods

### Variant discovery and genotyping workflow

Lancet2 builds on the previously described joint multi-sample colored de-Bruijn graph (cdbg)-based assembly framework [2]. As in the original Lancet approach, it processes the genome in consecutive, overlapping windows to perform read selection, graph construction, graph cleaning, active region detection, and path enumeration using an Edmond–Karp–style algorithm [24]. Building on this foundation, Lancet2 introduces additional modules for variant discovery, genotyping, and scoring.

**Figure 1.**
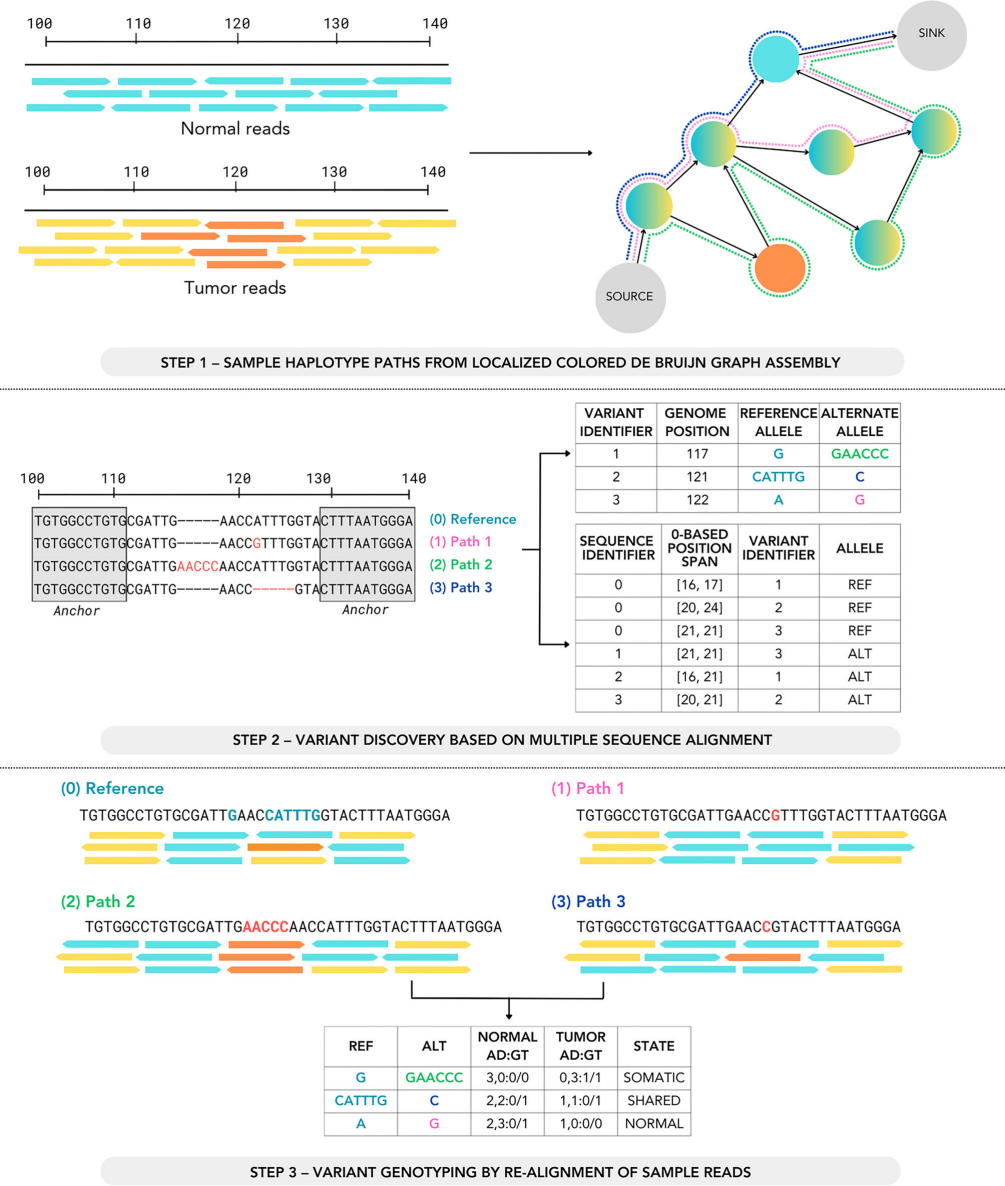
Illustration of various steps in the Lancet2 variant calling workflow. Step 1—Representation of tumor and normal reads aligned to a localized colored de Bruijn graph, showing sample haplotype paths derived from the graph’s structure. Step 2—Identification of candidate variants by performing partial order alignment-based MSAs of assembled contigs and the reference. Variants are listed with their genomic positions, reference alleles, and alternate alleles. Step 3—Genotyping of identified variants through the realignment of sample reads to the MSA, displaying normal and tumor allele depths, and genotypes for each variant path.

After assembling the local graph, Lancet2 aligns the assembled contigs and the corresponding reference sequence using a SIMD-accelerated partial order multiple sequence alignment (MSA) algorithm [25, 26]. MSA was performed using the partial order graphs strategy due to its efficiency and guaranteed optimal progressive MSA. Variants detected in these MSAs are recorded with their genomic coordinates, MSA positions, and reference/alternate alleles (Fig. 1, Step 2).

Subsequently, all constituent reads used in assembly are re-aligned to both the local reference and assembled contigs with the minimap2 library [27, 28]. Read alignments are then sorted by descending gap-compressed identity and alignment score before they are assigned as support using exact sequence match to the discovered variant alleles, ensuring each read contributes only once per variant. These support counts form the basis for genotype determination and allele frequency estimation in the sample.

### Building somatic glass-box boosting model

We trained the somatic glass-box boosting model using data from HCC1395 WGS_NS_T_1 (tumor) and WGS_NS_N_1 (normal) datasets. FASTQ files for both libraries were obtained from the Sequence Read Archive (SRA; ID: SRR7890893 for tumor and ID: SRR7890943 for normal) and processed through the NYGC cancer pipeline [4] to produce final alignments. Lancet2 was run on these tumor/normal pairs to generate a full set of raw candidate variants, which include germline, mosaic, and false-positive artifacts (non-somatic) in addition to somatic variants. These calls were annotated against the previously published high-confidence truth set for HCC1395 [23], labeling them as true somatic or non-somatic. Chromosome 1 variants were excluded for later testing.

Each candidate variant was characterized by a number of metrics/features, including window-level (percent high-quality reads per sample), variant-level (type, length, depth, absolute variant allele frequency (VAF) difference between tumor and normal), and allele-level (allele counts, strand bias, base quality, mapping quality) features, which are then utilized in the model training process. Due to an extreme class imbalance (∼1 true somatic per 200 non-somatic; see Supplementary Section 1 for more details), we applied random under-sampling to reduce the ratio to ~1:30. After under-sampling, we trained an Explainable Boosting Machine using the InterpretML framework [18]. The resulting trained model was saved to disk in Python’s native pickle format, enabling its direct integration into Lancet2’s filtering pipeline for somatic variant scoring. This integration greatly simplifies and improves the filtering process compared to multiple empirical hard cutoffs by relying on a single probabilistic score that can be used by the end user to balance precision and sensitivity.

### Enhanced “two-tech” truth set generation

The “two-tech” truth sets integrate short-read (Illumina) and long-read (Oxford Nanopore) variant calls, leveraging cross-technology validation to improve confidence and accuracy. For each of the four selected cancer cell lines, we began with the full output of the NYGC cancer pipeline [4] or the superSet calls [23] (short-read), and the PASS-filtered clairS v0.3.0 [29] variants (long read), intentionally including low-confidence calls to allow subsequent cross-validation. These call sets are compared using RTG vcfeval [30], and the resulting true positive variants from the short-read call set are included in the “two-tech” truth set as the “common” variants.

Beyond identifying variants present in both call sets, we attempted to “rescue” uniquely detected variants by performing a pileup-based genotyping step using Freebayes [31] in pooled continuous mode. Variants exclusive to one platform were included in the final “two-tech” truth set only if the alternate allele was supported by at least two reads at ≥20× coverage in the other platform data. For the HCC1395 cell line, we excluded all variants from chromosome arms 6p, 16q, and chrX because somatic and germline variants cannot be distinguished due to copy number loss in the normal sample [23]. Final variant intersections between short/long calls and the Freebayes calls were conducted using RTG vcfeval [30], ensuring that only cross-validated variants contributed to the enhanced truth sets.

### Architectural improvements

Lancet2 introduces several enhancements that streamline its architecture, improve interoperability, and increase performance. Following MSA, local assembly graphs can now be exported in GFA (Graphical Fragment Assembly) format, facilitating integration with a variety of external visualization and analysis tools (e.g. Bandage [32], VG [33], Sequence Tube Map [34]). Direct integration with htslib [35] allows Lancet2 to accept both BAM and CRAM alignment files, offering users flexibility in balancing storage and analysis constraints. Adopting modern C++20 language features ensures improved maintainability and testability over time. In addition, Lancet2 employs a pull-based, reactive multithreading model with a fast, lock-free concurrent queue [36]. This model dynamically distributes “work units” (genomic regions to process) across multiple worker threads, maximizing CPU utilization and optimizing performance throughout the entire runtime.

### Features and improvements to Sequence Tube Map

Several enhancements were introduced to Sequence Tube Map [34] to support visualization of Lancet2 variants using the exported GFA graphs along with aligned supporting reads. First, the input data selection system was generalized to handle multiple read “tracks” from different sources, requiring internal refactoring, new user interface components, and in-app documentation. Sequence Tube Map [34] can now load any number of distinct read tracks, along with graph or haplotype tracks, each displayed separately. This enables a clear distinction between tumor and normal read sets from Lancet2 output. A new dialog provides per-read details, including graph traversal paths.

To streamline loading Lancet2 outputs, support for pre-defined visualizations (“chunks”) was extended to let chunks carry their own track configuration, like UCSC Genome Browser sessions [37, 38]. These chunks can be fetched from remote servers using documented, testable URL formats, and users can navigate through them easily. This workflow integrates end-to-end with Lancet2’s prep_stm_viz.sh script, producing interactive visualizations directly from BAM/VCF inputs (see “Lancet Example” in the Sequence Tube Map demo server – https://vgteam.github.io/sequenceTubeMap). The full end-to-end workflow showing this integration between Lancet2 and Sequence Tube Map is available in Supplementary Section 5.

Finally, visualization drawing algorithms were refined to accommodate higher coverage and multiple BAM files. Node spacing was adjusted to ensure adequate horizontal space for each read path, and the coordinate bar now supports breaks due to these expansions. Read ordering logic was also improved to handle multiple tracks under complex topologies, including inversions and cycles.

## Results

### Outline of the Lancet2 workflow

Lancet2 builds upon the joint multi-sample colored de Bruijn graph assembly strategy used in Lancet [2], introducing new modules for variant discovery, genotyping, (Fig. 1) and filtering. Similar to the Lancet [2] tool, local haplotype contigs are generated by joint colored de Bruijn assembly of tumor and normal sample k-mers, through a process of graph clean-up, optimal k-mer size selection, and Edmond–Karp style path traversal [24]. To identify variants, Lancet2 performs Partial Order Alignment-based MSA [25, 26] of locally assembled contigs alongside the corresponding local reference sequence. Each sample read is subsequently re-aligned to the resulting MSA, ensuring that allele support is accurately determined by selecting the best-supported alleles for each read.

Following variant discovery and genotyping, candidate variants are then scored and filtered using an interpretable, machine learning–driven glass-box boosting model. This model was trained on the candidate Lancet2 variant calls (including germline and mosaic artifacts) annotated with high-confidence somatic truth set calls for the HCC1395 cell line [23] (Methods Section 2) using the InterpretML framework [16–18]. Unlike opaque black-box models, this approach enables researchers to easily comprehend why specific variants are classified as somatic, providing both fine-grained explanations at the individual variant level and broader insights into the model’s overall behavior (Supplementary Figs S1 and S2).

### Enhanced two-tech truth sets for cancer cell lines

**Figure 2.**
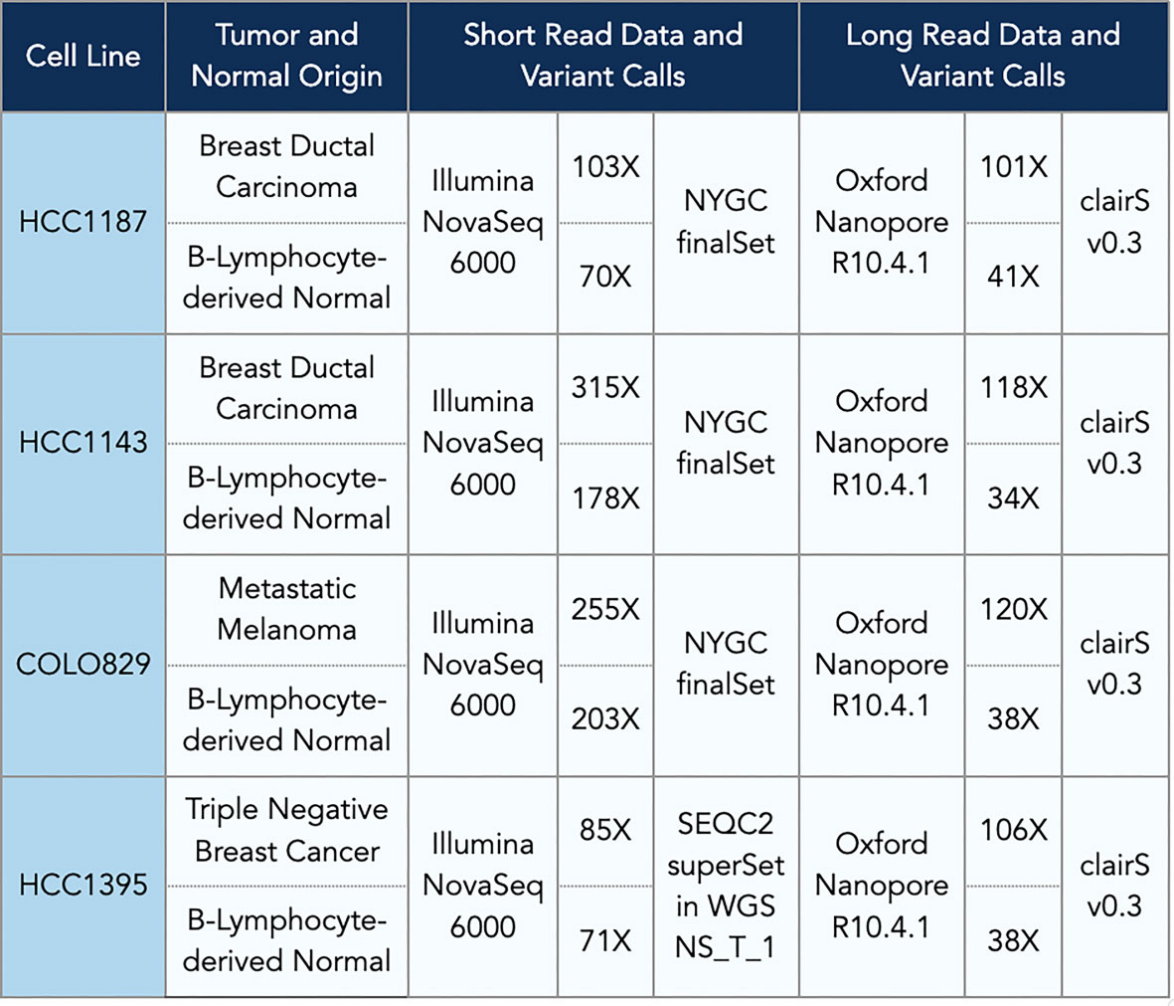
Details of four cancer cell lines, including tumor and normal sample origins, sequencing coverage, and variant call sets derived from short-read Illumina NovaSeq 6000 data and long-read Oxford Nanopore R10.4.1 data. Each row provides information for one cell line, listing the tumor type, normal sample origin, and corresponding coverage values for both sequencing technologies.

We integrated high-coverage short-read (Illumina) and long-read (Oxford Nanopore) data (Fig. 2) to enhance previously published benchmarking truth sets for four well-characterized cancer cell lines—HCC1187, HCC1143, COLO829, and HCC1395 [4, 23].

**Figure 3.**
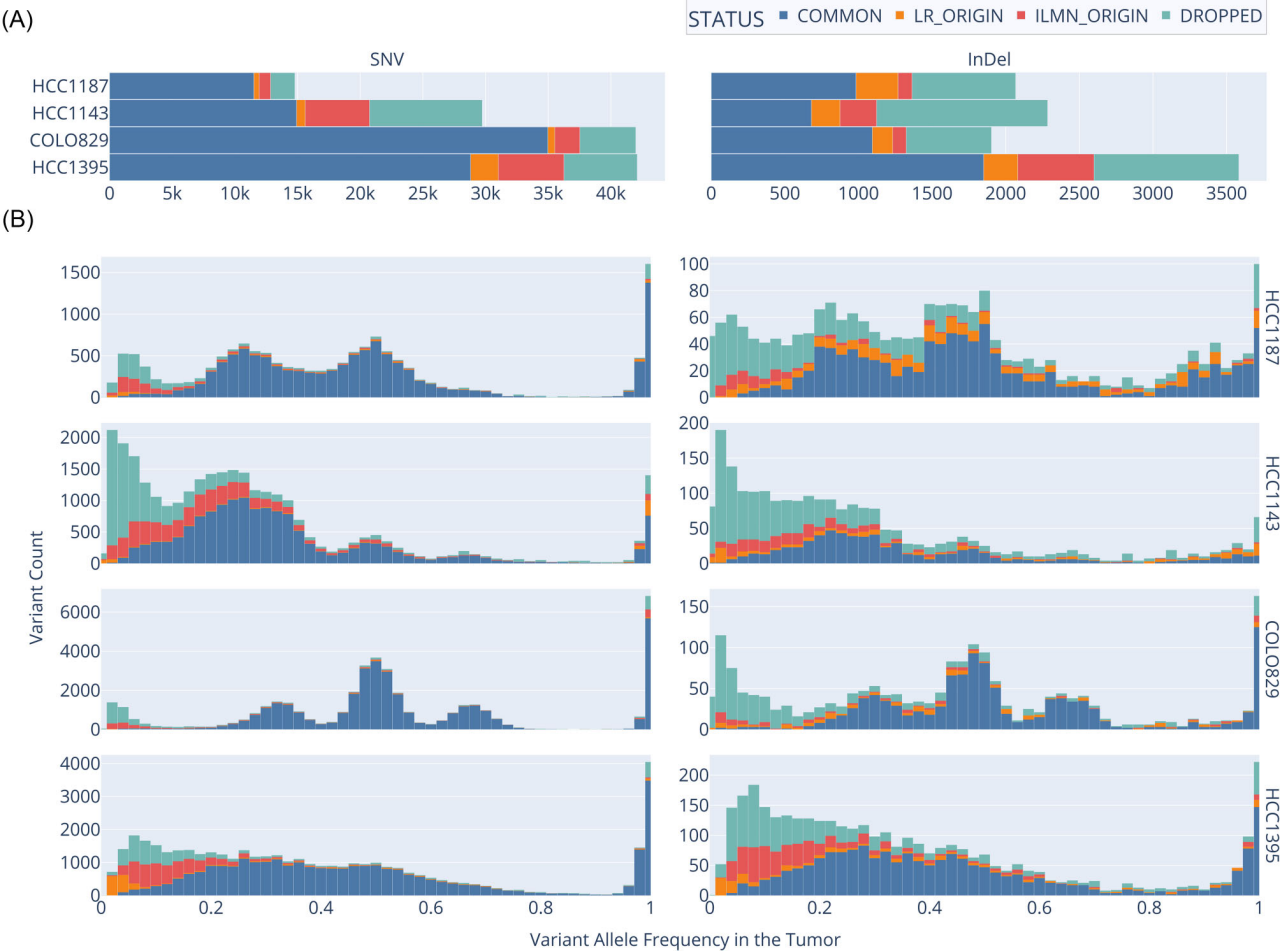
Visualization of the enhanced “two-tech” truth sets for four cancer cell lines (HCC1187, HCC1143, COLO829, and HCC1395), showing variant classifications and VAF distributions. (**A**) Stacked bar plots illustrating the counts of variants categorized as COMMON, LR_ORIGIN, ILMN_ORIGIN, and DROPPED for each cell line, grouped by variant type (Combined, SNV, InDel). (**B**) Histograms displaying the VAF distributions for each variant category and type, organized by cell line. Each row corresponds to a specific cell line, and each column presents the distribution of variants for SNV or InDel categories. Short-read data are used to extract the VAF for COMMON, ILMN_ORIGIN, and DROPPED variants. Long-read data are used to extract the VAF for LR_ORIGIN variants.

Beyond identifying variants supported by both platforms (COMMON), we systematically “rescued” variants initially observed by only one technology by examining raw alignments from the other platform (Supplementary Fig. S3). This approach yielded three additional variant categories (Fig. 3): variants called in long-read data and confirmed in short-read alignments (LR_ORIGIN), variants called in Illumina data and confirmed in long-read alignments (ILMN_ORIGIN), and variants without cross-platform evidence (DROPPED). Due to the lower sequencing coverage of the long-read datasets, some of the low VAF calls in Illumina are not likely to be confirmed by long-read data. However, variants called/missed in short/long read data due to technology specific mapping artifacts are greatly reduced because of this approach (Supplementary Figs S8–S23). The resulting “two-tech” truth sets represent a robust resource for community-wide benchmarking of somatic SNV and InDel variant callers.

### Variant calling performance evaluation

**Figure 4.**
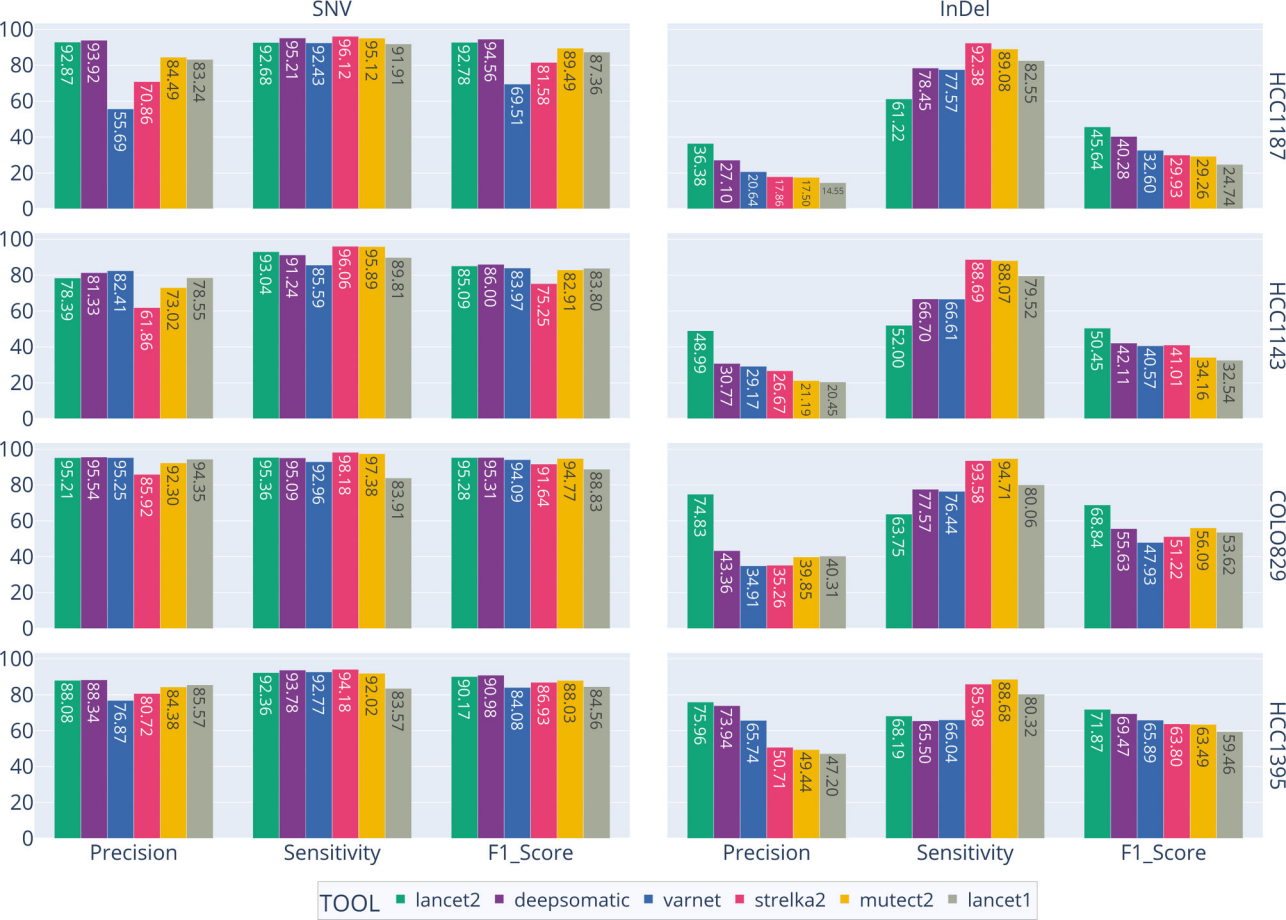
Comparison of variant calling performance metrics (precision, sensitivity, and F1 score) for multiple tools (Lancet2, DeepSomatic, VarNet, Strelka2, Mutect2, and Lancet1) benchmarked against the enhanced “two-tech” truth sets. Each panel represents a distinct variant type (SNV and InDel), and each row corresponds to one of four cancer cell lines (HCC1187, HCC1143, COLO829, and HCC1395). Bars indicate performance metrics for each tool, with numerical labels displayed.

Lancet2 achieves consistently higher InDel precision and F1 scores than competing variant callers across four distinct cancer cell lines (Fig. 4). However, for SNVs, DeepSomatic [21] outperforms Lancet2 in precision and F1 score when evaluated using our enhanced “two-tech” truth sets (Fig. 4). The default somatic model thresholds for Lancet2 are set at 95% probability for InDels and 90% for SNVs to ensure improved precision with a small impact on sensitivity. Users can fine-tune these cutoffs to prioritize higher sensitivity if their specific study demands it.

Notably, Lancet2 maintains its performance advantage when benchmarked against previously published high-confidence truth sets [4, 23], reinforcing the robustness of its improvements (Supplementary Fig. S4). Moreover, this accuracy advantage extends across the full VAF spectrum, particularly for InDels. In COLO829, e.g. other tools’ InDel calls suffer from elevated false-positive rates even at high VAFs, skewing their VAF profiles (Supplementary Fig. S5). By contrast, Lancet2 consistently delivers accurate InDel calls, underscoring its robust and reliable performance in diverse benchmarking scenarios.

### Runtime performance evaluation

**Figure 5.**
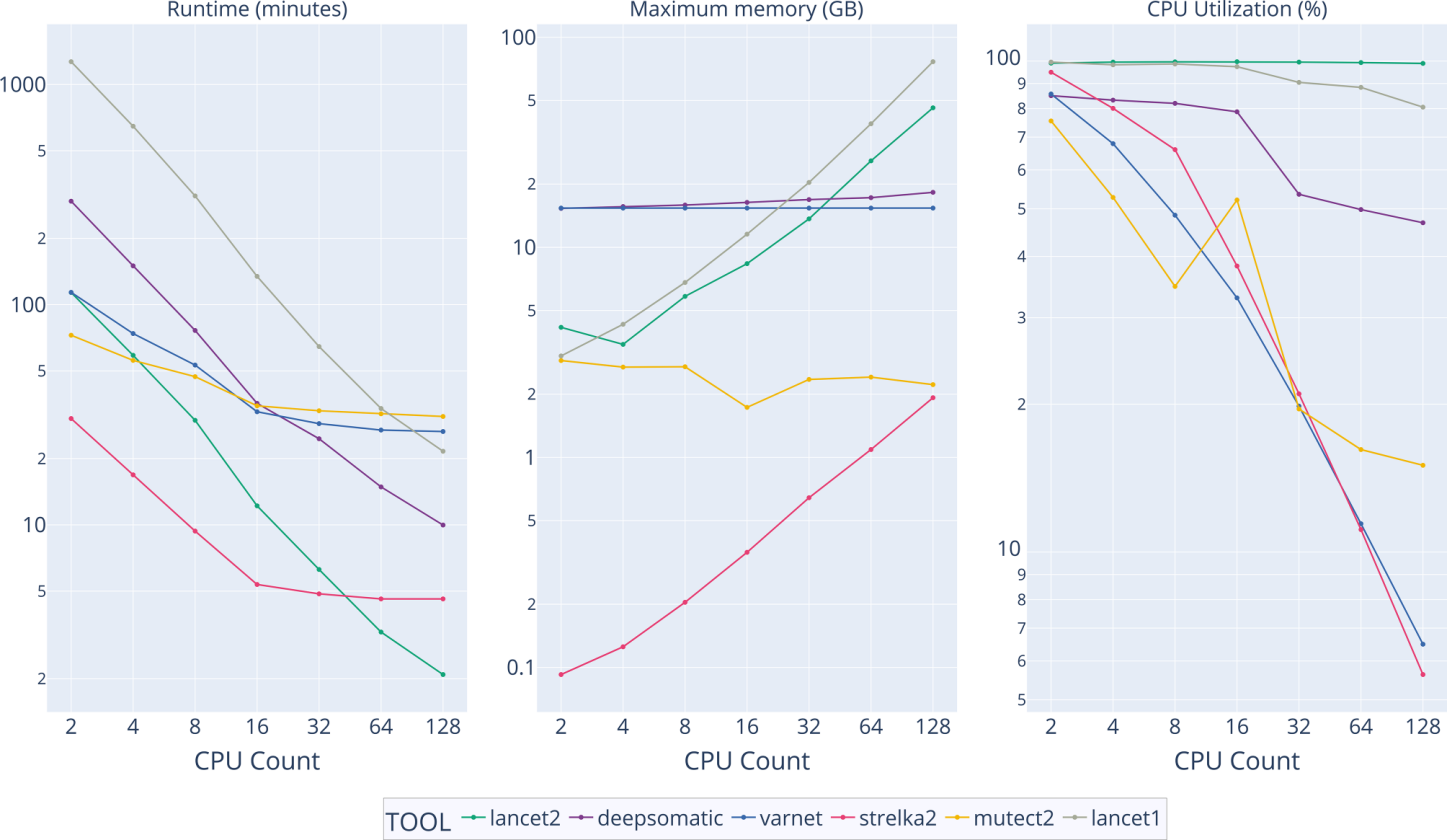
Runtime performance by number of CPUs: Benchmarked on Google Cloud with n2-highmem machines on chr20 HCC1395 versus HCC1395BL at 64× coverage. Line plots showing runtime in minutes, maximum memory usage in GB, and percent CPU utilization for variant calling tools (Lancet2, Lancet1, DeepSomatic, Mutect2, Strelka2, and VarNet) as the number of CPU cores increases. The *x*-axes represent increasing CPU counts, and the *y*-axes represent the respective metric values. Each line corresponds to a different tool.

Lancet2’s local assembly approach inherently increases computational demands compared to hybrid or alignment-based methods. Nonetheless, Lancet2 achieves approximately a 10-fold speed improvement over the original Lancet, and, in certain configurations, surpasses the performance of other tools due to its near-ideal scalability with additional CPU cores (Fig. 5). Memory usage is similarly optimized, showing a 40% reduction compared to Lancet1. This improvement becomes especially evident at higher coverage levels and when scaling to more cores (Supplementary Fig. S6). These results highlight Lancet2’s ability to leverage CPU resources efficiently across a range of sequencing coverages and hardware configurations while maintaining low memory-to-CPU ratios.

### Graph visualization of variants using Sequence Tube Map

**Figure 6.**
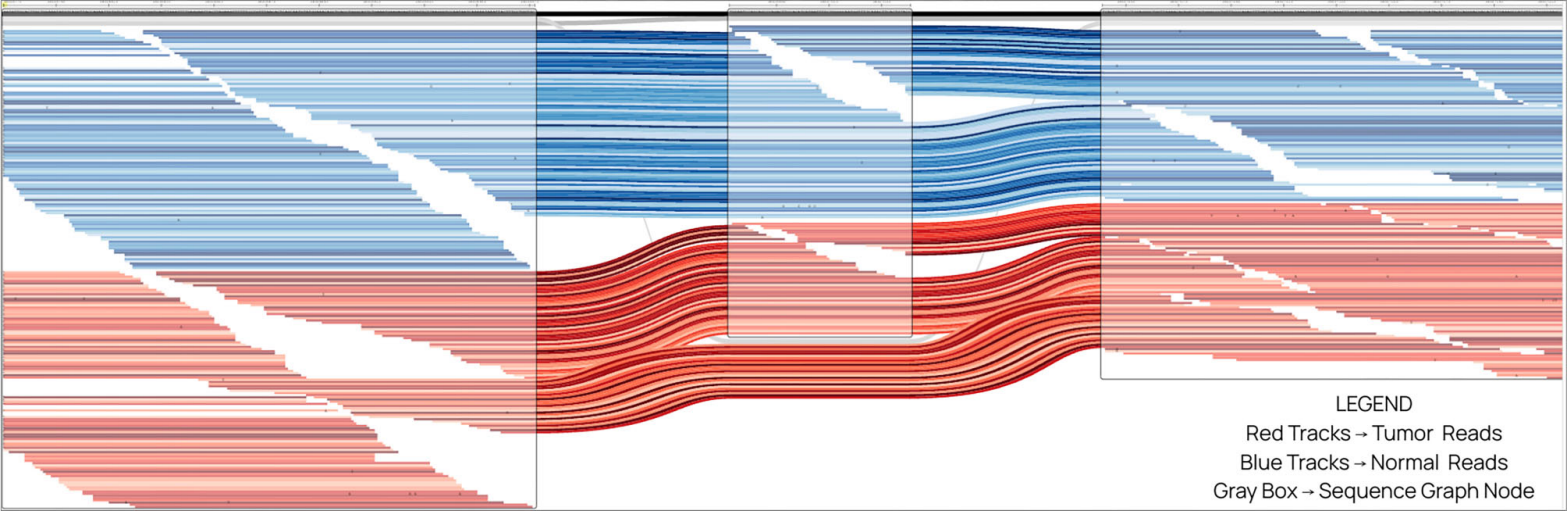
Sequence Tube Map visualization of a 70-bp somatic heterozygous deletion in HCC1395 tumor and normal cell lines with sample reads aligned to the local Lancet graph. The blue tracks correspond to normal sample reads, the red tracks below them correspond to tumor sample reads, and the gray boxes correspond to the nodes of the Lancet2’s exported sequence graph. The reads (red track) that skip the middle gray box confirm the presence of the deletion in the tumor sample.

Lancet2 addresses the longstanding challenge of visualizing complex variants, along with supporting reads from multiple samples in graph space, by its integration with Sequence Tube Map [34]. Users can easily export the MSAs used for variant discovery as GFA-formatted graphs, which are then directly loaded into the Sequence Tube Map environment (Supplementary Information). Once there, somatic variants and their supporting read alignments from multiple samples can be intuitively explored, enabling clinicians and researchers to confirm allele support, understand local genomic complexity, and gain insights into variant structures (Fig. 6 and Supplementary Fig. S7). This interoperability provides a user-friendly, interactive web interface for examining detected variants at a granular level, facilitating improved interpretation, verification, and communication in both research and clinical contexts.

## Discussion

We present Lancet2, a next-generation somatic variant caller that significantly improves Lancet1 in accuracy, efficiency, interpretability, and visualization. Our benchmarking results show that Lancet2 outperforms other industry-leading tools in InDel variant-calling performance, including Mutect2 [19], Strelka2 [20], VarNet [22], DeepSomatic [21], and the original Lancet [2], while maintaining competitive performance for SNVs. This aligns with previous studies highlighting the challenges of accurately identifying complex InDels using alignment-based approaches [2, 3], emphasizing the value of local-assembly methods in capturing subtle, structure-rich variants that may profoundly influence our understanding of tumor heterogeneity and clonal evolution.

A key advance in this work was the generation of enhanced benchmarking truth sets derived from integrating Illumina short-read and Oxford Nanopore long-read data for four commonly used cancer cell lines. By leveraging both sequencing platforms and “rescuing” variants identified by only one technology through cross-examination of raw alignments from the other, we produced a more robust and challenging “two-tech” truth set compared to traditional short-read-only truth sets (Supplementary Fig. S4 and Fig. 4 together show a general loss in sensitivity and precision across all tools when compared with the “two-tech” truth set). One limitation of the two-tech approach and the resulting truth set is the inability to confirm low-VAF variants due to the cost prohibitiveness of high-depth long-read sequencing. Nevertheless, this expanded resource represents a meaningful step forward, providing a more robust benchmark than purely short-read-based sets and enabling a more realistic evaluation of variant calling performance. As long-read sequencing becomes more pervasive, such integrative approaches to truth-set construction are increasingly recognized as critical for pushing the boundaries of somatic variant calling [23].

Beyond variant detection accuracy, Lancet2 demonstrates marked improvements in runtime and memory efficiency. Through careful algorithmic optimization and parallelization, Lancet2 is ~10 times faster and 40% more memory-efficient than the original Lancet, making it more practical for large-scale and cloud-based analyses. Notably, Lancet2’s nearly linear scalability with increasing CPU cores and its relatively stable resource consumption at higher coverage levels sets it apart from many existing tools. While previous studies have documented challenges in scaling variant callers to larger datasets and higher coverage [19, 20], our findings indicate that resource-efficient local assembly is achievable and can be integrated into routine pipelines without compromising accuracy. In fact, Lancet2’s modular nature and its speed improvements allow users to run pangenome style—multiple tumors, multiple normals joint calling—to generate raw candidate variants in joint VCF format. Future work is planned to simplify somatic variant analysis for longitudinal projects by integrating out-of-the-box custom filtering and annotation when using multi-sample (more than two samples) mode.

The improved interpretability of Lancet2’s somatic filtering, achieved by incorporating a transparent, intelligible machine learning model [18], is another important milestone. The ability to explain classification decisions both locally (for individual variants) and globally (across the full model) stands in contrast to more opaque “black box” filtering algorithms. This level of model transparency is increasingly valued in both research and clinical settings, where decision-making often requires scrutiny and validation of variant calls.

In addition, Lancet2 introduces graph-based visualization of cancer variants by its integration with Sequence Tube Map. This capability meets a pressing need to visualize both simple and complex variants and their supporting reads directly in graph space. Similar graph-centric approaches have been proposed in other genomic studies to better represent structural complexity and reduce reference bias [39, 40], but their adoption in routine somatic variant interpretation has lagged. This integration allows researchers and clinicians to intuitively navigate complex genomic regions, enhancing transparency and confidence in variant calls—particularly those that challenge conventional linear-reference paradigms.

Despite these advances, Lancet2 currently focuses on small variants, and broader applicability to larger structural events will require future work. Additionally, generating comprehensive two-tech truth sets demanded extensive data, underscoring the need for ongoing community efforts to produce standardized, robust benchmarks. Furthermore, the adaptability of Lancet2’s filtering model to entirely new sequencing technologies or cancer types has yet to be comprehensively assessed and may require retraining or calibration as new data types emerge.

In summary, Lancet2 represents a substantial step forward in the accuracy, efficiency, and interpretability of somatic variant calling. Its performance improvements over industry-leading methods and its validated robustness across multiple well-characterized cancer cell lines suggest that it can serve as a valuable tool in both research and clinical genomics workflows.

## Supplementary Material

lqag036_Supplemental_Files

## Data Availability

Lancet2 is an open-source software distributed under a BSD 3-Clause License at https://github.com/nygenome/Lancet2 (https://doi.org/10.5281/zenodo.19190601). The workflows and scripts to reproduce the benchmarking analysis performed for this manuscript can be obtained at the following bitbucket repository – https://bitbucket.nygenome.org/projects/COMPBIO-INTERNAL/repos/lancet2_manuscript/browse (https://doi.org/10.5281/zenodo.19190426). Short-read Illumina NovaSeq and long-read Oxford Nanopore PromethION alignment files for the four cancer cell lines and their matched normals used in the “two-tech” truth set generation process are available at the following SRA accession IDs. Short read Illumina data—COLO829 (SRX6743552), COLO829BL (SRX6743554), HCC1187 (SRX6743560), HCC1187BL (SRX6743562), HCC1143 (SRX6743556), HCC1143BL (SRX6743558), HCC1395 (SRX4728475), and HCC1395BL (SRX4728425). Long-read Oxford Nanopore data—COLO829 (SRX27516919), COLO829BL (SRX27516920), HCC1187 (SRX27516921), HCC1187BL (SRX27516922), HCC1143 (SRX27516923), HCC1143BL (SRX27516924), HCC1395 (SRX27516925), and HCC1395BL (SRX27516926). The resulting “two-tech” truth set variant call files (VCF), along with the original short-read-only truth sets for the individual cell lines, are available at the following link—https://console.cloud.google.com/storage/browser/lancet2-paper/truths (https://doi.org/10.5281/zenodo.19191073).

## References

[1] Narzisi G, Schatz MC. The challenge of small-scale repeats for indel discovery. Front Bioeng Biotechnol. 2015;3:8. https://www.frontiersin.org/journals/bioengineering-and-biotechnology/articles/10.3389/fbioe.2015.00008. 25674564 10.3389/fbioe.2015.00008 PMC4306302

[2] Narzisi G, Corvelo A, Arora K et al. Genome-wide somatic variant calling using localized colored de Bruijn graphs. Commun Biol. 2018;1:20. 10.1038/s42003-018-0023-9. 30271907 PMC6123722

[3] Köster J, Dijkstra LJ, Marschall T et al. Varlociraptor: enhancing sensitivity and controlling false discovery rate in somatic indel discovery. Genome Biol. 2020;21:98. 10.1186/s13059-020-01993-6. 32345333 PMC7187499

[4] Arora K, Shah M, Johnson M et al. Deep whole-genome sequencing of 3 cancer cell lines on 2 sequencing platforms. Sci Rep. 2019;9:19123. 10.1038/s41598-019-55636-3. 31836783 PMC6911065

[5] Widman AJ, Shah M, Frydendahl A et al. Ultrasensitive plasma-based monitoring of tumor burden using machine-learning-guided signal enrichment. Nat Med. 2024;30:1655–66. 10.1038/s41591-024-03040-4. 38877116 PMC7616143

[6] Yamada M, Keller RR, Gutierrez RL et al. Childhood cancer mutagenesis caused by transposase-derived PGBD5. Sci Adv. 2025;10:eadn4649. 10.1126/sciadv.adn4649. PMC10959420 38517960

[7] Khani F, Hooper WF, Wang X et al. Evolution of structural rearrangements in prostate cancer intracranial metastases. NPJ Precis Oncol. 2023;7:91. 10.1038/s41698-023-00435-3. 37704749 PMC10499931

[8] Nguyen DD, Hooper WF, Liu W et al. The interplay of mutagenesis and ecDNA shapes urothelial cancer evolution. Nature. 2024;635:219–28. 10.1038/s41586-024-07955-3. 39385020 PMC11541202

[9] Lozada JR, Geyer FC, Selenica P et al. Massively parallel sequencing analysis of benign melanocytic naevi. Histopathology. 2019;75:29–38. 10.1111/his.13843. 30791119 PMC6752735

[10] Sebastiao APM, Pareja F, Kumar R et al. Genomic analysis of recurrences and high-grade forms of polymorphous adenocarcinoma. Histopathology. 2019;75:193–201. 10.1111/his.13854. 30843621 PMC7920221

[11] Selenica P, Raj N, Kumar R et al. Solid pseudopapillary neoplasms of the pancreas are dependent on the Wnt pathway. Mol Oncol. 2019;13:1684–92. 10.1002/1878-0261.12490. 30972907 PMC6670010

[12] Basturk O, Weigelt B, Adsay V et al. Sclerosing epithelioid mesenchymal neoplasm of the pancreas—a proposed new entity. Mod Pathol. 2020;33:456–67. 10.1038/s41379-019-0334-5. 31383964 PMC7000300

[13] Poulos RC, Perera D, Packham D et al. Scarcity of recurrent regulatory driver mutations in colorectal cancer revealed by targeted deep sequencing. JNCI Cancer Spectr. 2019;3:pkz012. 10.1093/jncics/pkz012. 31360895 PMC6649856

[14] Marchiò C, Da Cruz Paula A, Gularte-Merida R et al. PAX8–GLIS3 gene fusion is a pathognomonic genetic alteration of hyalinizing trabecular tumors of the thyroid. Mod Pathol. 2019;32:1734–43. 10.1038/s41379-019-0313-x. 31273314 PMC7442035

[15] Yi D, Xu L, Luo J et al. Germline TP53 and MSH6 mutations implicated in sporadic triple-negative breast cancer (TNBC): a preliminary study. Hum Genomics. 2019;13:4. 10.1186/s40246-018-0186-y. 30630526 PMC6327518

[16] Lou Y, Caruana R, Gehrke J. Intelligible models for classification and regression. In: Proceedings of the 18th ACM SIGKDD International Conference on Knowledge Discovery and Data Mining. KDD ’12. Association for Computing Machinery, 2012, 150–8. 10.1145/2339530.2339556.

[17] Lou Y, Caruana R, Gehrke J et al. Accurate intelligible models with pairwise interactions. In: Proceedings of the 19th ACM SIGKDD International Conference on Knowledge Discovery and Data Mining. KDD ’13. Association for Computing Machinery, 2013, 623–31. 10.1145/2487575.2487579.

[18] Nori H, Jenkins S, Koch P et al. InterpretML: a unified framework for machine learning interpretability. arXiv, https://arxiv.org/abs/1909.09223, 9 September 2019, preprint: not peer reviewed.

[19] Benjamin D, Sato T, Cibulskis K et al. Calling somatic SNVs and indels with Mutect2. bioRxiv, 10.1101/861054, 2 December 2019, preprint: not peer reviewed.

[20] Kim S, Scheffler K, Halpern AL et al. Strelka2: fast and accurate calling of germline and somatic variants. Nat Methods. 2018;15:591–4. 10.1038/s41592-018-0051-x. 30013048

[21] Park J, Cook DE, Chang PC et al. Accurate somatic small variant discovery for multiple sequencing technologies with DeepSomatic. Nat Biotechnol. 2025. 10.1038/s41587-025-02839-x. 41102444

[22] Krishnamachari K, Lu D, Swift-Scott A et al. Accurate somatic variant detection using weakly supervised deep learning. Nat Commun. 2022;13:4248. 10.1038/s41467-022-31765-8. 35869060 PMC9307817

[23] Fang LT, Zhu B, Zhao Y et al. Establishing community reference samples, data and call sets for benchmarking cancer mutation detection using whole-genome sequencing. Nat Biotechnol. 2021;39:1151–60. 10.1038/s41587-021-00993-6. 34504347 PMC8532138

[24] Edmonds J, Karp RM. Theoretical improvements in algorithmic efficiency for network flow problems. J ACM. 1972;19:248–64. 10.1145/321694.321699.

[25] Lee C, Grasso C, Sharlow MF. Multiple sequence alignment using partial order graphs. Bioinformatics. 2002;18:452–64. 10.1093/bioinformatics/18.3.452. 11934745

[26] Vaser R, Sović I, Nagarajan N et al. Fast and accurate *de novo* genome assembly from long uncorrected reads. Genome Res. 2017;27:737–46. 10.1101/gr.214270.116. 28100585 PMC5411768

[27] Li H . Minimap2: pairwise alignment for nucleotide sequences. Bioinformatics. 2018;34:3094–100. 10.1093/bioinformatics/bty191. 29750242 PMC6137996

[28] Li H . New strategies to improve minimap2 alignment accuracy. Bioinformatics. 2021;37:4572–4. 10.1093/bioinformatics/btab705. 34623391 PMC8652018

[29] Zheng Z, Su J, Chen L et al. ClairS: a deep-learning method for long-read somatic small variant calling. bioRxiv, 10.1101/2023.08.17.553778, 21 August 2023, preprint: not peer reviewed.

[30] Cleary JG, Braithwaite R, Gaastra K et al. Comparing variant call files for performance benchmarking of next-generation sequencing variant calling pipelines. bioRxiv, 10.1101/023754, 2 August 2015, preprint: not peer reviewed.

[31] Garrison E, Marth G. Haplotype-based variant detection from short-read sequencing. arXiv, https://arxiv.org/abs/1207.3907, 20 July 2012, preprint: not peer reviewed.

[32] Wick RR, Schultz MB, Zobel J et al. Bandage: interactive visualization of *de novo* genome assemblies. Bioinformatics. 2015;31:3350–2. 10.1093/bioinformatics/btv383. 26099265 PMC4595904

[33] Garrison E, Sirén J, Novak AM et al. Variation graph toolkit improves read mapping by representing genetic variation in the reference. Nat Biotechnol. 2018;36:875–9. 10.1038/nbt.4227. 30125266 PMC6126949

[34] Novak A, Chung D, Sundar S. Sequence Tube Map. GitHub. https://github.com/vgteam/sequenceTubeMap (24 October 2025, date last accessed).

[35] Bonfield JK, Marshall J, Danecek P et al. HTSlib: c library for reading/writing high-throughput sequencing data. Gigascience, 2021;10:giab007. 10.1093/gigascience/giab007. 33594436 PMC7931820

[36] Cameron. GitHub. https://github.com/cameron314/concurrentqueue (24 October 2025, date last accessed).

[37] Kent WJ, Sugnet CW, Furey TS et al. The human genome browser at UCSC. Genome Res. 2002;12:996–1006. 10.1101/gr.229102. 12045153 PMC186604

[38] Perez G, Barber GP, Benet-Pages A et al. The UCSC Genome Browser database: 2025 update. Nucleic Acids Res. 2025;53:D1243–9. 10.1093/nar/gkae974. 39460617 PMC11701590

[39] Iqbal Z, Caccamo M, Turner I et al. *De novo* assembly and genotyping of variants using colored de Bruijn graphs. Nat Genet. 2012;44:226–32. 10.1038/ng.1028. 22231483 PMC3272472

[40] Paten B, Novak AM, Eizenga JM et al. Genome graphs and the evolution of genome inference. Genome Res. 2017;27:665–76. 10.1101/gr.214155.116. 28360232 PMC5411762

