# Supplementary material for: Lancet2: Improved and accelerated somatic variant calling with joint multi-sample local assembly graphs
Source: NAR Genom Bioinform. 2026 Apr 7;8(2):lqag036. doi: 10.1093/nargab/lqag036 (PMC13064521; doi:10.1093/nargab/lqag036)
Supplement: lqag036_Supplemental_Files [file lqag036_supplemental_files.zip › Lancet2_Supplementary_final.pdf]

Supplementary Information

1. Somatic glass box boosting model

Lancet2 v2.8.4-main-6ef7ba445a was used with default parameters to call all raw variants with alternate allele support greater than at least 2 reads from the HCC1395 vs HCC1395BL NovaSeq libraries obtained from SRA (SRR7890893 for tumor and SRR7890943 for normal) and processed through the NYGC cancer pipeline[4]. RTG vcfeval[34] was then used to compare the raw Lancet2 variants with the previously published high-confidence truth set for HCC1395[5], resulting in the classification of the raw variants into 7,798,060 false positive variants missing in the truth set and 39,249 true positive variants present in the truth set.

```
Lancet2 pipeline --num-threads 224 \
--reference GRCh38_full_analysis_set_plus_decoy_hla.fa \
--out-vcfgz HCC1395.Lancet_v2.8.4-main-6ef7ba445a.vcf.gz \
--normal HCC1395BL_SAMN10102574_SRR7890943.bam \
--tumor HCC1395_SAMN10102573_SRR7890893.bam

rtg vcfeval --output-mode=annotate --template \
GRCh38_full_analysis_set_plus_decoy_hla.sdf \
--all-records --vcf-score-field QUAL --sample="ALT,ALT" \
--evaluation-regions SEQC2_High-Confidence_Regions_v1.2.bed \
--baseline "SEQC2.high-Confidence_Combined.v1.2.1.vcf.gz" \
```

```
--calls HCC1395.Lancet_v2.8.4-main-6ef7ba445a.vcf.gz \
--output rtg_vcfeval_output_HCC1395
```

All the variants from chromosome 1 were left out in the training process to be used in the later testing/benchmarking process. Due to the extreme class imbalance between the false vs true positive sets, the false positive variants were randomly undersampled to pick only one million variants. The full list of all the features that were extracted into a data frame for 34,176 true positive somatic variants and 1,000,000 false positive non-somatic variants are available in Supplementary file 1 (`somatic_EBM_model_features.txt`).

Explainable Boosting Classifier from InterpretML[19] v0.5.1 was then used to train the model with parameters max_bins=32, smoothing_rounds=2000, max_rounds=25000. The resulting somatic glass box boosting model that was built is accessible publicly at https://storage.googleapis.com/lancet-ml-models/somatic_ebm.lancet_6ef7ba445a.v1.pkl. Global term/feature importances of the somatic glass box model can be explored (Supplementary Figure 1) using the following snippet of python code.

```
import pickle
import interpret

with open("somatic_ebm.lancet_6ef7ba445a.v1.pkl", "rb") as rf:
    model = pickle.load(rf)
    interpret.show(model.explain_global())
```

Local explanation for why a single variant is classified as somatic/non-somatic can also be explored (Supplementary Figure 2) using the following python code snippet.

```
interpret.show(model.explain_local(X, Y))
```

This enables end users and researchers to provide a definitive answer to why a particular variant was marked as somatic or non-somatic by the machine learning model.

2. Enhanced Two-Tech Truth set generation

For the HCC1187, HCC1143 and COLO829 cell lines, previously published NYGC v6 cancer pipeline final calls [4,5] (includes lower confidence calls with support from a single variant caller as well) were used as the short read call set. For the HCC1395 cell line, v1.2 superSet calls from the Sequencing Quality Control Phase 2 Consortium [4,5] (SEQC2) were used as the short read call set. Long read data for the 4 cancer cell lines were generated on the Oxford Nanopore R10 flow cell. Prior to variant calling, each sample dataset was base called using `dorado v0.7.1_80da5f5` with the v4.1.0 super high accuracy (sup) model followed by read alignment to GRCh38 reference genome using `minimap2 v2.28-r1209`. PASS calls from `clairS v0.3.0` were then used as the long read call set.

The clairS command line used to generate per chromosome variant calls for the long read call set is as follows –

```
/usr/bin/time --verbose /opt/bin/run_clairs \
      --tumor_bam_fn ~{tumorCram} \
      --normal_bam_fn ~{normalCram} \
      --ref_fn ~{referenceFasta} \
      --platform ont_r10_dorado_sup_4khz \
      --enable_indel_calling --ctg_name ~{contigName} \
      --sample_name ~{tumorSampleName} --threads 64 \
      --output_dir "$(pwd)" \
      --output_prefix "~{outFilePrefix}.snv" \
      --indel_output_prefix "~{outFilePrefix}.indel"
```

To generate the "two-tech" truth set, RTG vcfeval v3.12.1 was used to intersect the short read and long read call sets. The RTG command line used to generate the intersection is shown below.

```
rtg vcfeval \
--template GRCh38_full_analysis_set_plus_decoy_hla.sdf \
--baseline ${SHORT_READ_TRUTH_SET_CALLS} \
--calls ${LONG_READ_CLAIRS_PASS_CALLS} \
--all-records --sample=ALT,ALT --vcf-score-field=QUAL \
--output-mode=split --output LR_vs_SR_output
```

The variants that are common between both the short and long read call sets form the initial set of “two-tech” call set. We then attempted to rescue the variants that are uniquely seen either in the short or long read call sets into the “two-tech” truth set by inspecting for evidence of variants in the raw alignments, using `Freebayes v1.3.8` in pooled continuous mode. The command line that was used to inspect for evidence of variants in long or short read alignments is as follows –

```
# Attempt to rescue variants uniquely present
# in the short read call set into “two-tech” calls
# by looking for them in long read alignments
/usr/bin/time --verbose freebayes \
--fasta-reference GRCh38_full_analysis_set_plus_decoy_hla.fa \
--variant-input ${SHORT_READ_CALLSET_UNIQUE_VCF} \
--targets ${SHORT_READ_CALLSET_UNIQUE_REGIONS_BED} \
--only-use-input-alleles --hwe-priors-off \ --binomial-obs-
priors-off --allele-balance-priors-off \
--no-population-priors --legacy-gls \
--pooled-continuous --pooled-discrete \
--min-alternate-fraction 0 --min-alternate-count 1 \
--exclude-unobserved-genotypes \
${LONG_READ_TUMOR_BAM} ${LONG_READ_NORMAL_BAM}

# Attempt to rescue variants uniquely present
```

```
# in the long read call set into "two-tech" calls
# by looking for them in short read alignments
/usr/bin/time --verbose freebayes \
--fasta-reference GRCh38_full_analysis_set_plus_decoy_hla.fa \
--variant-input ${LONG_READ_CALLSET_UNIQUE_VCF} \
--targets ${LONG_READ_CALLSET_UNIQUE_REGIONS_BED} \
--only-use-input-alleles --hwe-priors-off \ --binomial-obs-
priors-off --allele-balance-priors-off \
--no-population-priors --legacy-gls \
--pooled-continuous --pooled-discrete \
--min-alternate-fraction 0 --min-alternate-count 1 \
--exclude-unobserved-genotypes \
${SHORT_READ_TUMOR_BAM} ${SHORT_READ_NORMAL_BAM}
```

The variants that were "rescued" using Freebayes are then filtered for sufficient evidence using the following `bcftools` (`v1.20`) expression, before they are added into the "two-tech" call set.

```
# At least 20x depth in both tumor and normal
# At least 2 or more reads supporting ALT allele in tumor
# No reads supporting ALT allele in normal
--include 'FMT/DP[0]>=20 && FMT/DP[1]>=20 && SUM(FMT/AO[0:*])>=2
&& SUM(FMT/AO[1:*])==0'
```

The entire workflow for “two-tech” truth set generation starting from the intersection of the long read and short read variant call sets is shown in Supplementary Figure 3.

The IGV screenshots (Supplementary Figures 8 to 23) containing both short and long read alignments from COLO829 tumor & normal samples show various examples of different classes of variants from the “two-tech” truth set generation process –

- Common (variants that are common between the short and long read call sets)
- LR_Origin (variants only in the long read call set validated by short read)
- ILMN_Origin (variants only in the short read call set validated by long read)
- Dropped (variants dropped from previously published high confidence truth set)

3. Variant Calling Performance Evaluation

The full command lines and exact tool versions that were used to run each of the variant callers on a single chromosome for performance evaluation are as follows –

Lancet1 v1.1.0_b6c9067

```
set -euxo pipefail

/usr/bin/time --verbose lancet \
      --num-threads "~{numCpus}" \
      --normal "~{normalBamCram}" \
```

```
        --tumor "~{tumorBamCram}" \
        --ref "~{referenceFasta}" \
        --reg "~{contigRegion}" \
        --max-avg-cov 1000 \
        > tmp.lancet1_out.vcf 2> "stderr.~{outputPrefix}.log" \
&& bcftools reheader \
        --fai "~{referenceFaidx}" "tmp.lancet1_out.vcf" \
| bcftools view -Oz -o "~{outputPrefix}.vcf.gz" /dev/stdin \
&& bcftools index --tbi "~{outputPrefix}.vcf.gz"

Lancet2 v2.8.5-main-affb044c
set -euxo pipefail

# Increase max open files limit
ulimit -n 16384; ulimit -a

/usr/bin/time -v Lancet2 pipeline \
        --normal "~{normalBamCram}" \
        --tumor "~{tumorBamCram}" \
        --num-threads "~{numCpus}" \
        --reference "~{referenceFasta}" \
        --region "~{contigRegion}" \
        --out-vcfgz "~{outputPrefix}.vcf.gz" \
        |& tee "stderr.~{outputPrefix}.log"
```

```
DeepSomatic v17_rc0_07012024
set -euxo pipefail

/usr/bin/time --verbose run_deepsomatic \
      --model_type=WGS --ref="~{referenceFasta}" \
      --reads_normal="~{normalBamCram}" \
      --reads_tumor="~{tumorBamCram}" \
      --sample_name_tumor=~{tumorName} \
      --sample_name_normal=~{normalName} \
      --num_shards="~{numCpus}" \
      --regions="~{contigRegion}" \
      --output_vcf="~{outputPrefix}.vcf.gz" \
      |& tee "stderr.~{outputPrefix}.log"

Mutect2 gatk-v4.6.0.0
set -euxo pipefail

# Mutect2 needs ref dict and exact sample name in RG header
gatk CreateSequenceDictionary --REFERENCE "~{referenceFasta}"

gatk GetSampleName -I "~{normalBamCram}" -O normalName.txt && \
gatk GetSampleName -I "~{tumorBamCram}" -O tumorName.txt
```

```
JAVA_OPTS="-Xmx~{memoryInGb}g -XX:ParallelGCThreads=~{numCpus}"

/usr/bin/time --verbose gatk Mutect2 \
      --java-options ”~{JAVA_OPTS}” \
      --native-pair-hmm-threads "~{numCpus}" \
      --input "~{normalBamCram}" --input "~{tumorBamCram}" \
      --reference "~{referenceFasta}" \
      --intervals "~{contigRegion}" \
      --normal-sample "$(cat normalName.txt)" \
      --tumor-sample "$(cat tumorName.txt)" \
      --output "~{outputPrefix}.raw.vcf.gz" \
      --annotation-group StandardMutectAnnotation \
      |& tee "stderr.~{outputPrefix}.log"

/usr/bin/time --verbose gatk FilterMutectCalls \
      --java-options ”~{JAVA_OPTS}” \
      --reference "~{referenceFasta}" \
      --variant "~{outputPrefix}.raw.vcf.gz" \
      --output "~{outputPrefix}.vcf.gz" \
      |& tee "stderr.~{outputPrefix}.log"
```

Strelka2 v2.9.10-manta-v1.6.0

```
set -euxo pipefail
```

```
226
227 mkdir -p "~{outputPrefix}.manta_runDir" \
228           "~{outputPrefix}.strelka_runDir"
229
230 chromRegex="^~{contigRegion}\t"
231
232 printf "%s\t0\t%s\n" \
233         "~{contigRegion}" \
234         "$(grep -P ${chromRegex} ~{referenceFaidx} | cut -f2)" \
235 | bgzip -c >| call_regions.bed.gz \
236 && tabix -p bed call_regions.bed.gz
237
238 configManta.py \
239       --referenceFasta "~{referenceFasta}" \
240       --normalBam "~{normalBamCram}" \
241       --tumourBam "~{tumorBamCram}" \
242       --runDir "manta_runDir" \
243       --callRegions "call_regions.bed.gz" \
244 && /usr/bin/time --verbose \
245       "manta_runDir/runWorkflow.py" \
246       --mode local --jobs "~{numCpus}" \
247       |& tee "stderr.~{outputPrefix}.log"
248
249 configureStrelkaSomaticWorkflow.py \
```

```
250         --referenceFasta "~{referenceFasta}" \
251         --normalBam "~{normalBamCram}" \
252         --tumourBam "~{tumorBamCram}" \
253         --runDir "strelka_runDir" \
254         --callRegions "call_regions.bed.gz" \
255         --indelCandidates \
256   "manta_runDir/results/variants/candidateSmallIndels.vcf.gz" \
257   && /usr/bin/time --verbose \
258         "strelka_runDir/runWorkflow.py" \
259         --mode local --jobs "~{numCpus}" \
260         |& tee "stderr.~{outputPrefix}.log"
261
262   Varnet v1.1.0
263   set -euxo pipefail
264
265   mkdir -p "~{outputPrefix}"
266   chromRegex="^~{contigRegion}\t"
267
268   printf "%s\t0\t%s\n" \
269          "~{contigRegion}" \
270          "$(grep -P ${chromRegex} ~{referenceFaidx} | cut -f2)" \
271          >| region.bed
272
273   /usr/bin/time --verbose python /VarNet/filter.py \
```

```
        --processes "~{numCpus}" \
        --sample_name "~{tumorName}" \
        --reference "~{referenceFasta}" \
        --region_bed "region.bed" \
        --normal_bam "~{normalBamCram}" \
        --tumor_bam "~{tumorBamCram}" \
        --output_dir "~{outputPrefix}" \
        |& tee "stderr.~{outputPrefix}.log" \
&& /usr/bin/time --verbose python /VarNet/predict.py \
        --processes "~{numCpus}" \
        --sample_name "~{tumorName}" \
        --reference "~{referenceFasta}" \
        --normal_bam "~{normalBamCram}" \
        --tumor_bam "~{tumorBamCram}" \
        --output_dir "~{outputPrefix}" \
        |& tee "stderr.~{outputPrefix}.log"
```

The per chromosome variant calls from each caller was then normalized and combined using the following bcftools (v1.20) commands

```
# Normalize & Combine VCFs
bcftools reheader --fai "~{referenceFaidx}" ~{rawVcfGz} \
| bcftools norm -Oz -o "~{outputVcf}" \
```

```
            --fasta-ref "~{referenceFasta}" --check-ref s \
&& bcftools index --tbi "~{outputVcf}" \
&& bcftools concat -Ou ~{sep=' ' vcfs} \
| bcftools sort -m12G -Oz -o "~{outputPrefix}.vcf.gz" \
&& bcftools index --tbi "~{outputPrefix}.vcf.gz"
```

`Java v1.9` and `RTG vcfeval v3.12.1` were then used to benchmark each variant caller against the truth set using the following command –

```
rtg vcfeval \
    --template GRCh38_full_analysis_set_plus_decoy_hla.sdf \
    --baseline ${TRUTH_VCF} \
    --calls ${CALLSET_VCF} \
    --output ${OUTPUT_DIRECTORY} \
    --all-records --sample=ALT,ALT \
    --vcf-score-field=QUAL \
    --output-mode=annotate
```

The full workflow used to perform variant caller performance evaluation which also contains additional scripts to postprocess tool VCFs and generate an interactive benchmarking HTML report is available at the following git repository – https://bitbucket.nygenome.org/projects/COMPBIO-INTERNAL/repos/lancet2_manuscript/browse.

For convenience the interactive benchmarking reports generated from the analysis are available at the following links –

Using the Two-Tech Truth set

https://storage.googleapis.com/lancet2-paper/reports/vs_two_tech_truth/HCC1187/HCC1187_WholeGenome.html

https://storage.googleapis.com/lancet2-paper/reports/vs_two_tech_truth/HCC1143/HCC1143_WholeGenome.html

https://storage.googleapis.com/lancet2-paper/reports/vs_two_tech_truth/COLO829/COLO829_WholeGenome.html

https://storage.googleapis.com/lancet2-paper/reports/vs_two_tech_truth/HCC1395/HCC1395_WholeGenome.html

Using the previously published Illumina only High Confidence Truth set

https://storage.googleapis.com/lancet2-paper/reports/vs_ilmn_hc_truth/HCC1187/HCC1187_WholeGenome.html

https://storage.googleapis.com/lancet2-paper/reports/vs_ilmn_hc_truth/HCC1143/HCC1143_WholeGenome.html

https://storage.googleapis.com/lancet2-paper/reports/vs_ilmn_hc_truth/COLO829/COLO829_WholeGenome.html

https://storage.googleapis.com/lancet2-paper/reports/vs_ilmn_hc_truth/HCC1395/HCC1395_WholeGenome.html

4. Runtime Performance Evaluation

The same command lines were used to perform runtime performance evaluation for all the variant callers as shown previously in supplementary section 3. Two different benchmarking experiments were performed on Google Cloud using HCC1395 tumor and matched normal (HCC1395BL) Illumina datasets with the following variables – 1.) constant coverage dataset with increasing CPU core counts. 2.) constant CPU core count with increasing dataset coverage. To study the scalability of variant callers, we performed the first benchmarking experiment on n2-highmem machines with varying CPU core count (2, 4, 8, 16, 32, 64, 128 CPU cores were used) to process chromosome 20 of HCC1395 tumor and matched normal data, both down sampled to 64x coverage. To study the impact of sample coverage, the second benchmarking experiment was performed on n2-highmem-128 machines with increasing coverage (64x/64x, 128x/128x, 256x/256x, 512x/512x, 1024x/1024x tumor/normal coverages were used) to process chromosome 20 of HCC1395 tumor and matched normal data.

5. Graph Visualization of Variants using Sequence Tube Map

The following steps describe the workflow to visualize Lancet2 variants of interest in graph space using the Sequence Tube Map framework.

I. Install prerequisite tools – Lancet2 (https://github.com/nygenome/Lancet2), samtools, bcftools, vg version 1.59.0, and jq, and ensure that they are available as commands that can be executed in the environment PATH.

II. Install Sequence Tube Map (https://github.com/vgteam/sequenceTubeMap) version `0452ecb82d057372e359a9b456d789336e5ab8a1`.

III. Use the Lancet2 `prep_stm_viz.sh` script to run Lancet2 on a small set of variants of interest that need to be visualized in the Sequence Tube Map. The script will run Lancet2 using the `--graph-dir` flag to generate GFA formatted sequence graphs for each variant of interest. Local VG graphs and indices required to load the sample reads along with the Lancet2 graph are then constructed, enabling simplified use with the "custom" data option in the Sequence Tube Map interface.

IV. After running the Sequence Tube Map Server as detailed in the Tube Map Readme, set "Data" to "custom" and "BED file" to "index.bed". Pick a "Region" (or variant) of interest and hit "Go" to visualize.

Sequence Tube Map demo server containing somatic variants from COLO829 tumor/normal pair sample – https://shorturl.at/JQZsy

Supplementary Figures

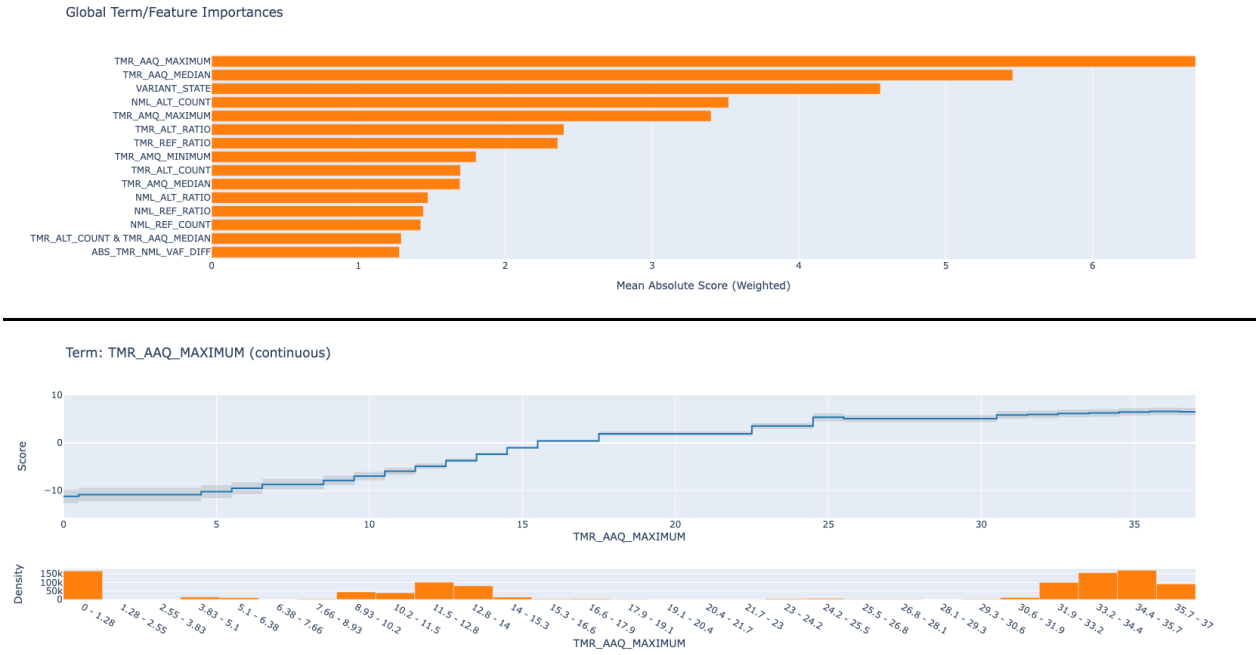

Supplementary Figure 1: Global Explanation of the Somatic Glass box Model. The first panel shows the relative terms/feature importances and their mean weighted scores learned by the Somatic Glass box model. The second panel shows the expected feature response curve for a single feature in the model (TME_AAQ_MAXIMUM). The X axis shows the values of the feature in question with the density histogram showing its distribution in the training set. The Y axis represents the log probability score learned by the model at a particular X-axis value.

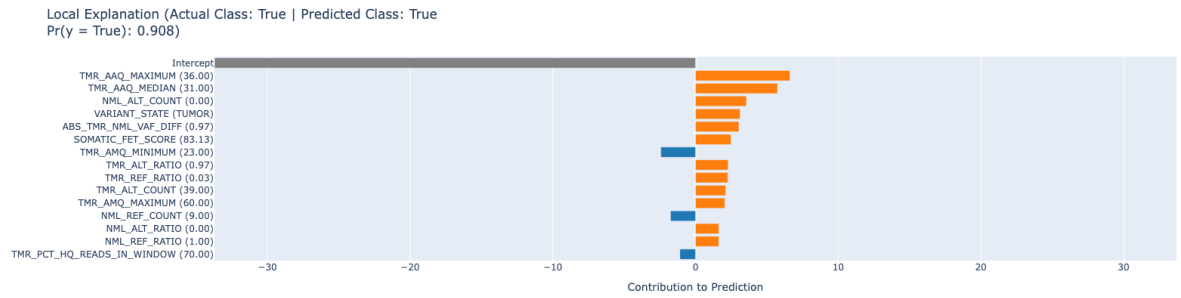

382

383 **Supplementary Figure 2: Local Explanation of the Somatic Glass box Model. Shows the**

384 **individual contribution of the most important features used by the model to arrive at**

385 **its final prediction and probability value for the classification given a single variant.**

386 **The feature values of the variant in question are shown in brackets along the Y-axis.**

387

388

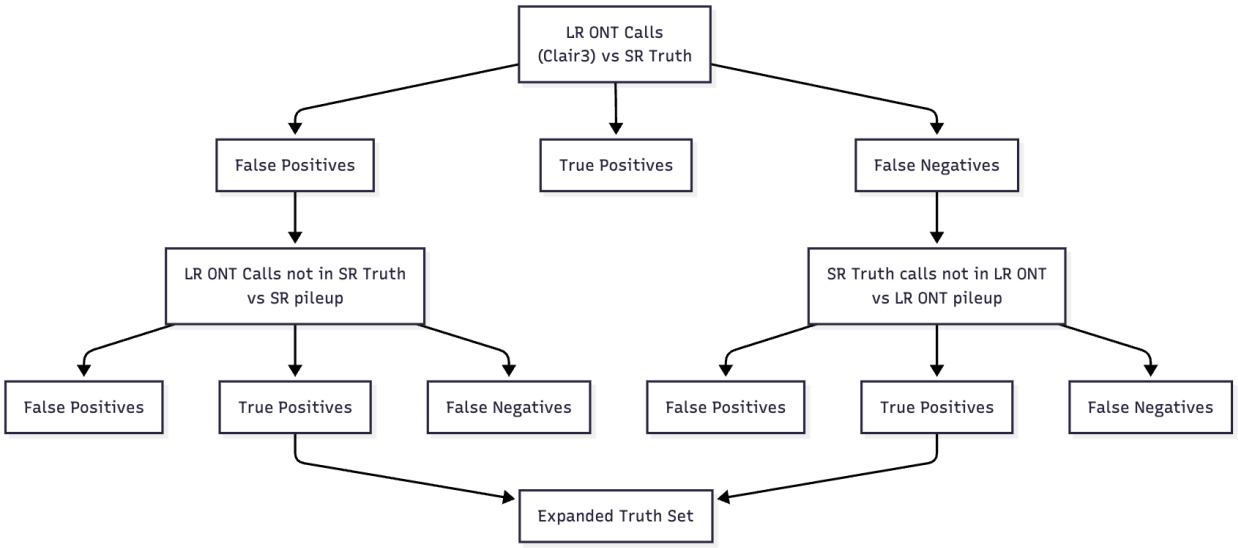

389

390 **Supplementary Figure 3: Flowchart showing a high-level overview of the process used**

391 **to “rescue” variants unique to a single technology into the “two-tech” truth set. All**

392 **intersections between two different call sets were performed using RTG vcfeval**

v3.12.1. The terms “true positives”, “false positives” and “false negatives” refer to the output VCFs generated by vcfeval in the split output mode.

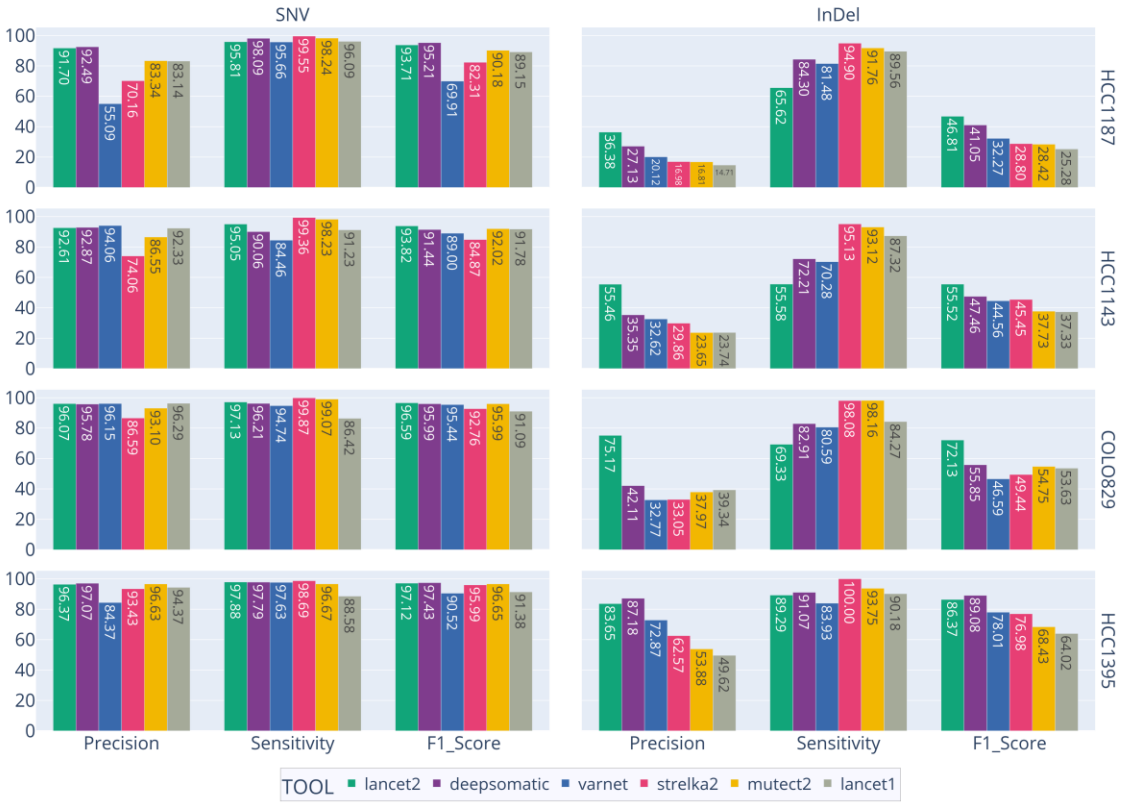

Supplementary Figure 4: Comparison of variant calling performance metrics (precision, sensitivity, and F1 score) for multiple tools (Lancet2, DeepSomatic, VarNet, Strelka2, Mutect2, Lancet1) benchmarked against previously published high-confidence truth sets. Each panel represents a distinct variant type (SNV, InDel), and each row corresponds to one of four cancer cell lines (HCC1187, HCC1143, COLO829, HCC1395). Bars indicate the performance metric values for each tool, with numerical labels displayed.

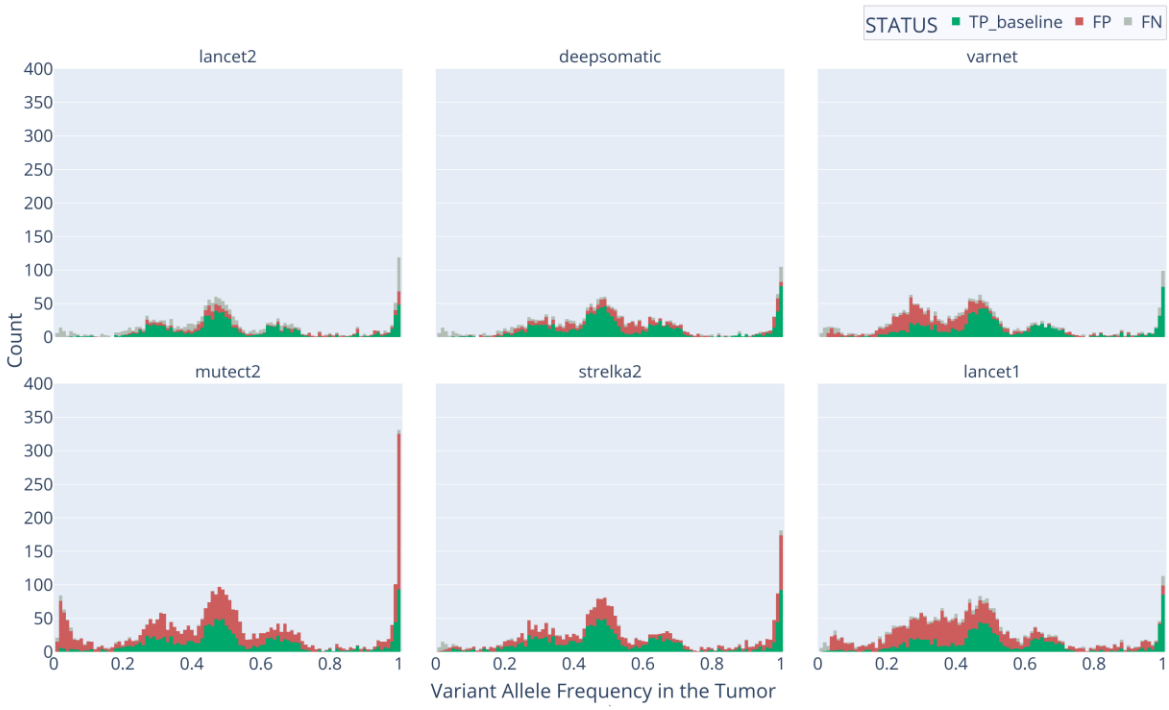

Supplementary Figure 5: Variant allele frequency (VAF) distributions of true positive calls, false positives, and false negatives for InDel variants identified by multiple variant callers (Lancet2, DeepSomatic, VarNet, Strelka2, Mutect2, and Lancet1) in the COLO829 dataset. Each panel shows the count of variants at different VAF levels stratified by call status for a single calling tool. VAF is extracted from the truth set calls for false negative calls and extracted from the variant caller output for true positive and false positive calls.

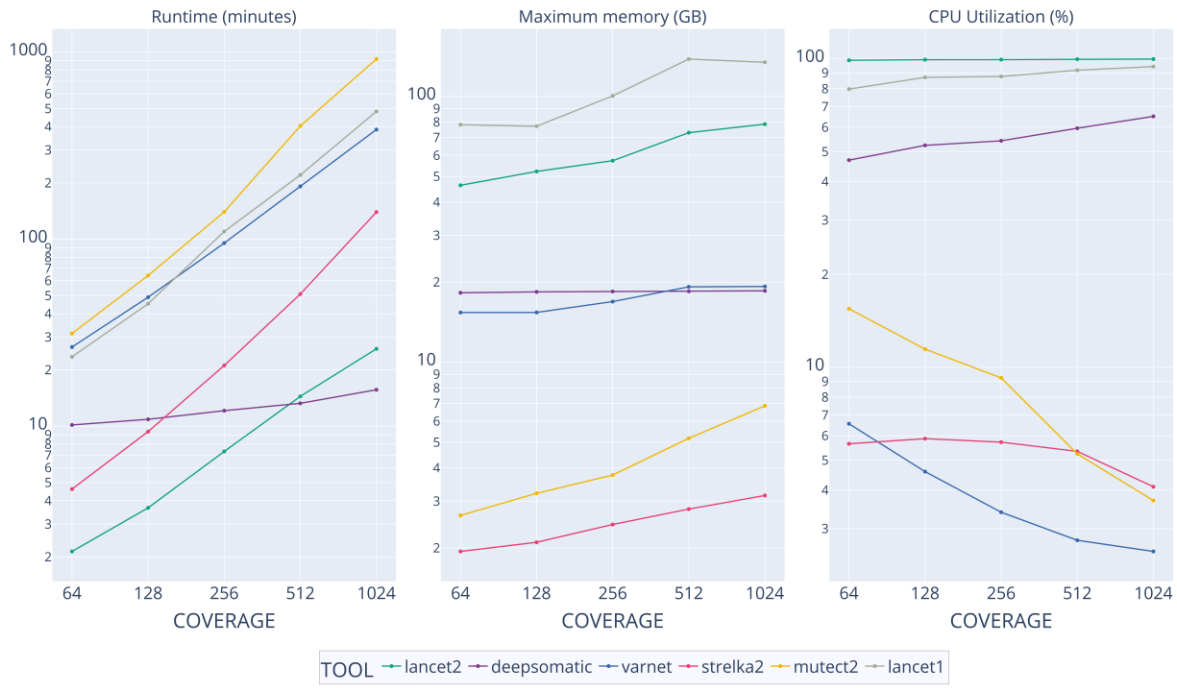

411

412 **Supplementary Figure 6: Runtime Performance by Coverage: Benchmarked on Google**

413 **Cloud with a 128 core (n2-highmem-128) machine on chr20 HCC1395 vs HCC1395BL**

414 **with the same coverage. Three line plots showing runtime in minutes, maximum**

415 **memory usage in GB, and percent CPU utilization for multiple variant calling tools**

416 **(Lancet2, Lancet1, DeepSomatic, Mutect2, Strelka2, VarNet) as sequencing coverage**

417 **increases. The x-axes represent increasing coverage levels, and the y-axes represent**

418 **the respective metric values. Each line corresponds to a different tool.**

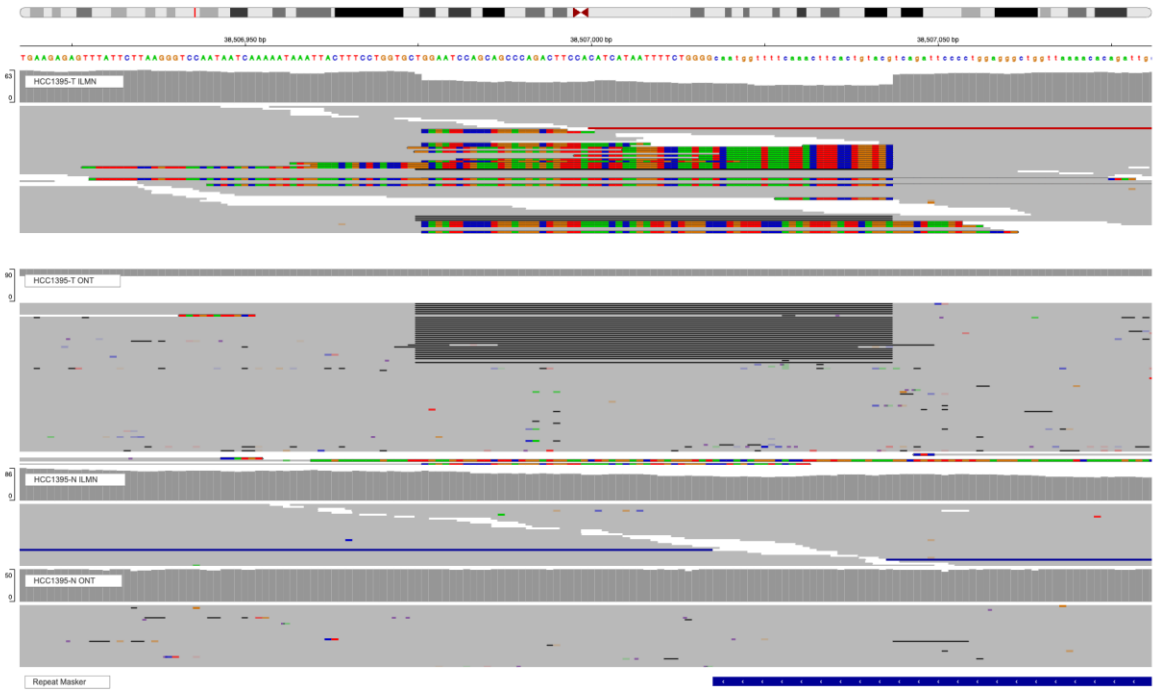

Supplementary Figure 7: Integrated Genome Viewer (IGV) visualization of the same 70-bp somatic deletion in HCC1395 tumor and normal cell lines as in Figure 5 with alignment tracks for short read tumor, long read tumor, short read normal and long read normal samples respectively.

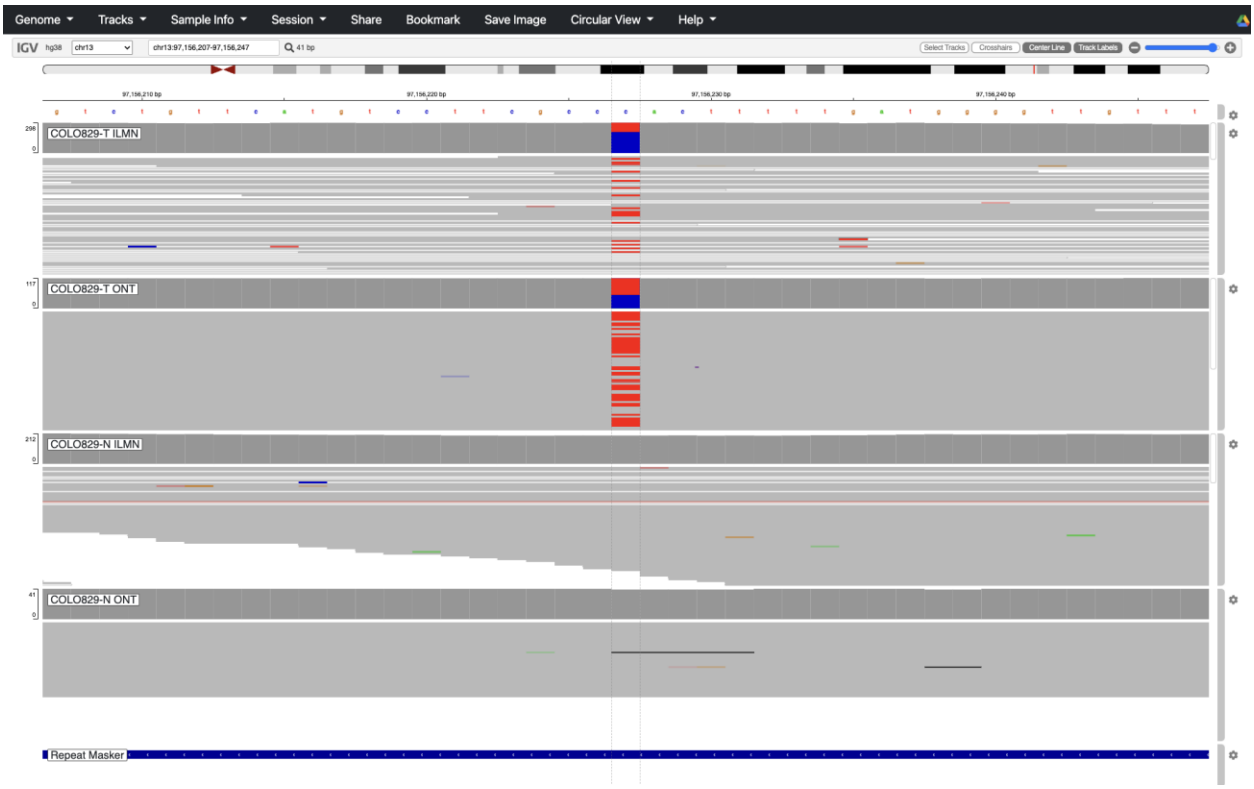

Supplementary Figure 8: C → T somatic SNV in COLO829 at chr13:97156227 common between the short and long read call sets.

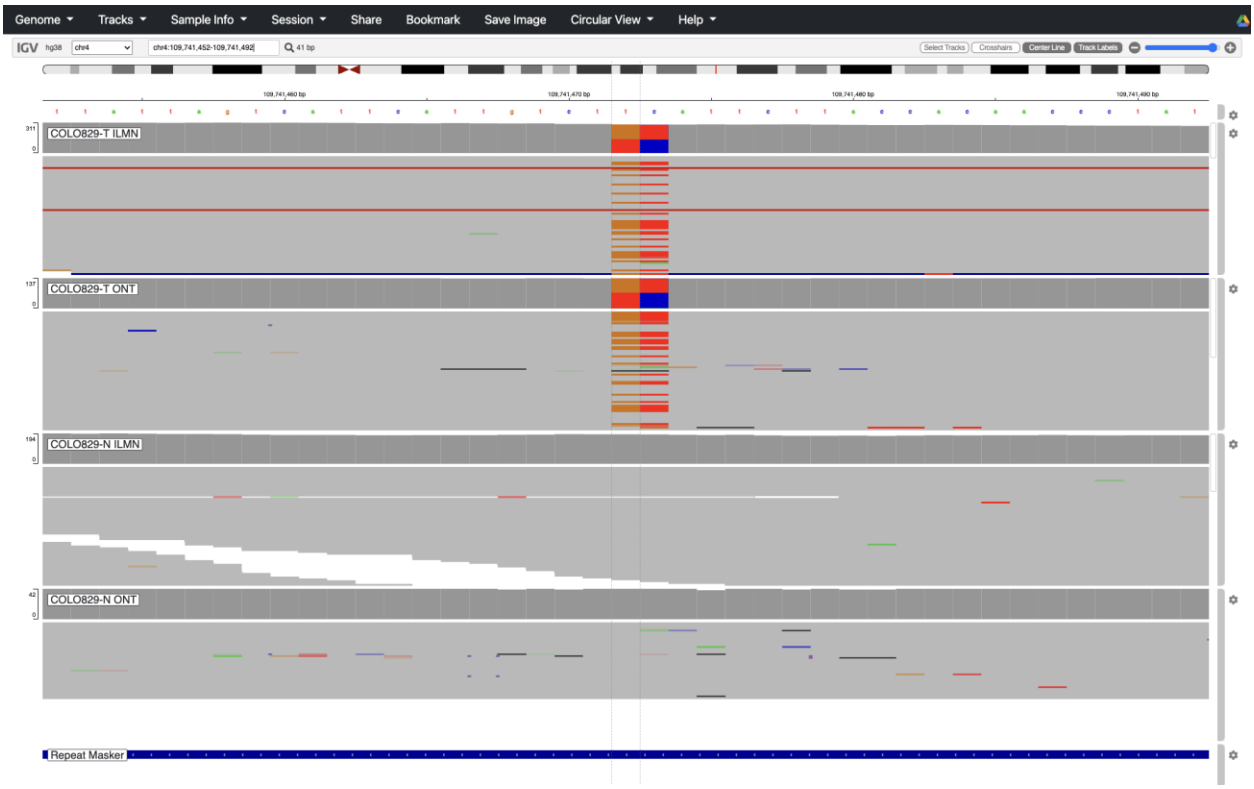

Supplementary Figure 9: TC → GT somatic MNV in COLO829 at chr4:109741472 common between the short and long read call sets.

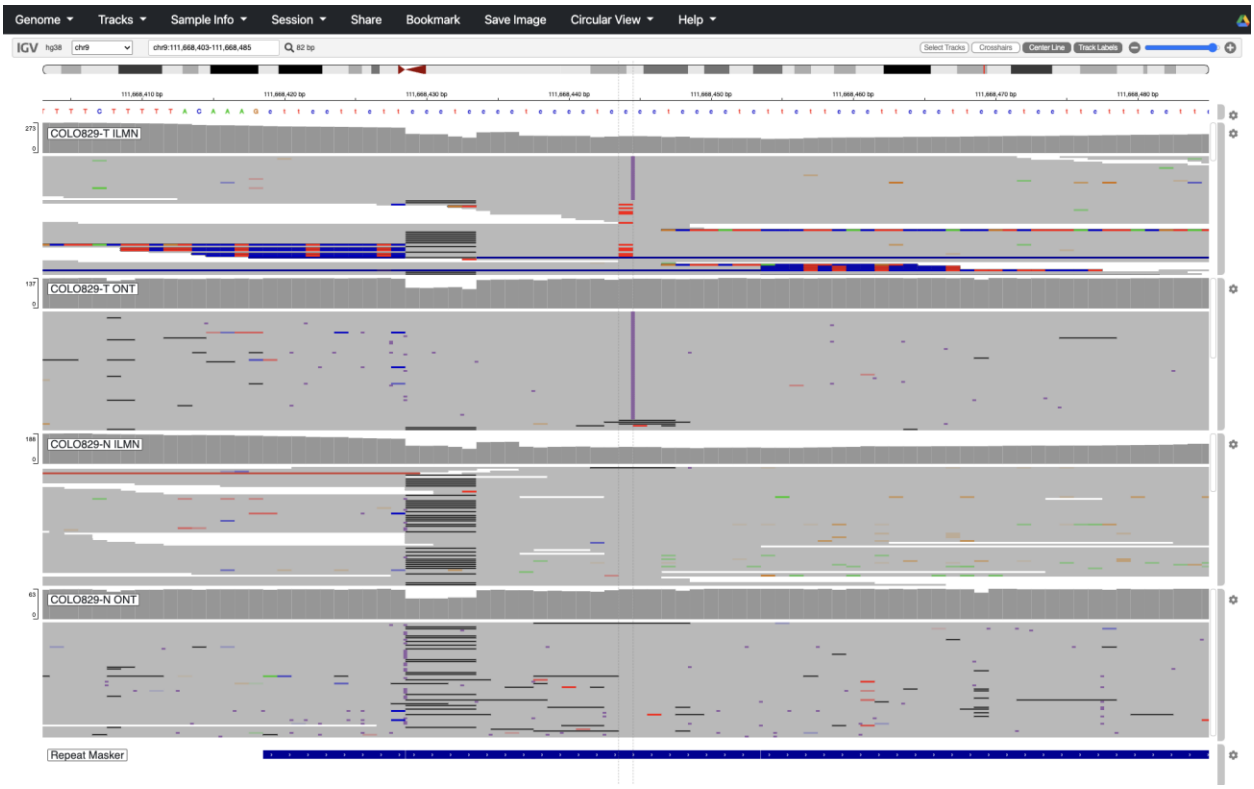

Supplementary Figure 10: 15 bp somatic INS in COLO829 at chr9:111668444 common between the short and long read call sets.

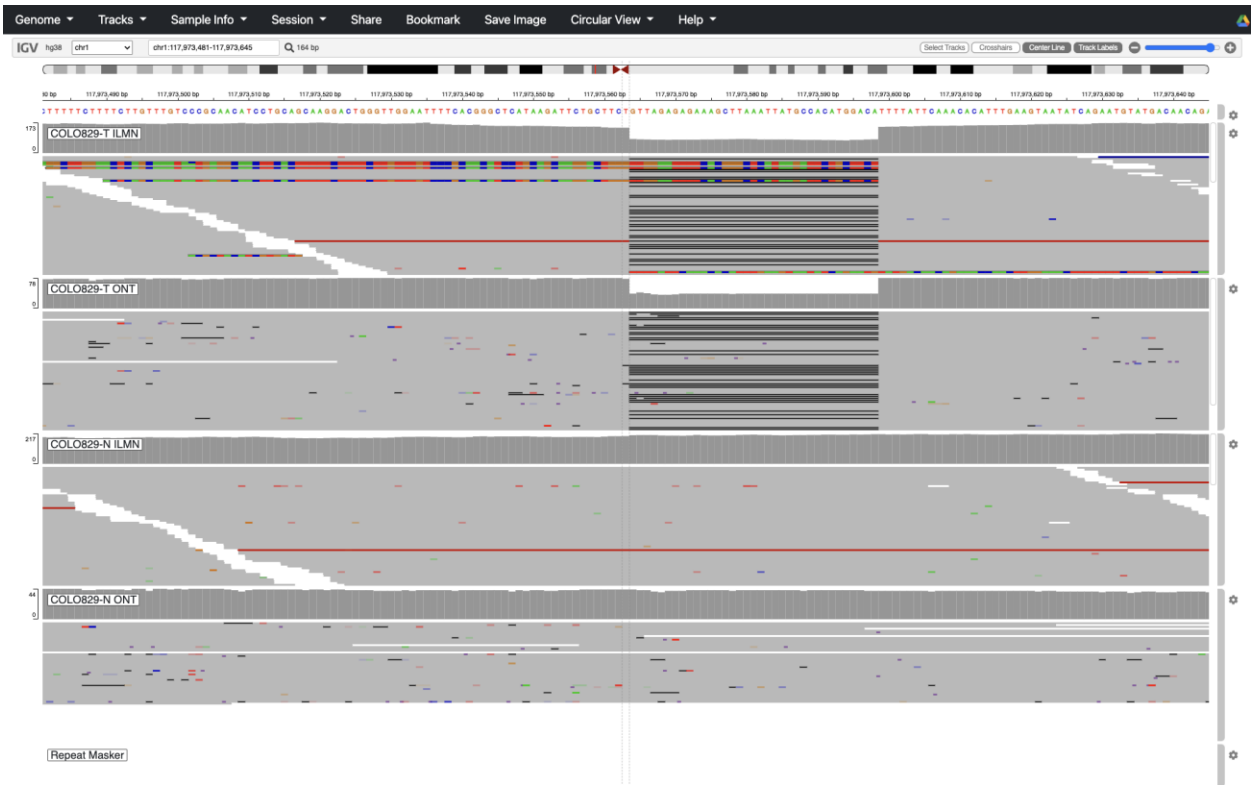

433

434 **Supplementary Figure 11: 35 bp somatic DEL in COLO829 at chr1:117973563 common**

435 **between the short and long read call sets.**

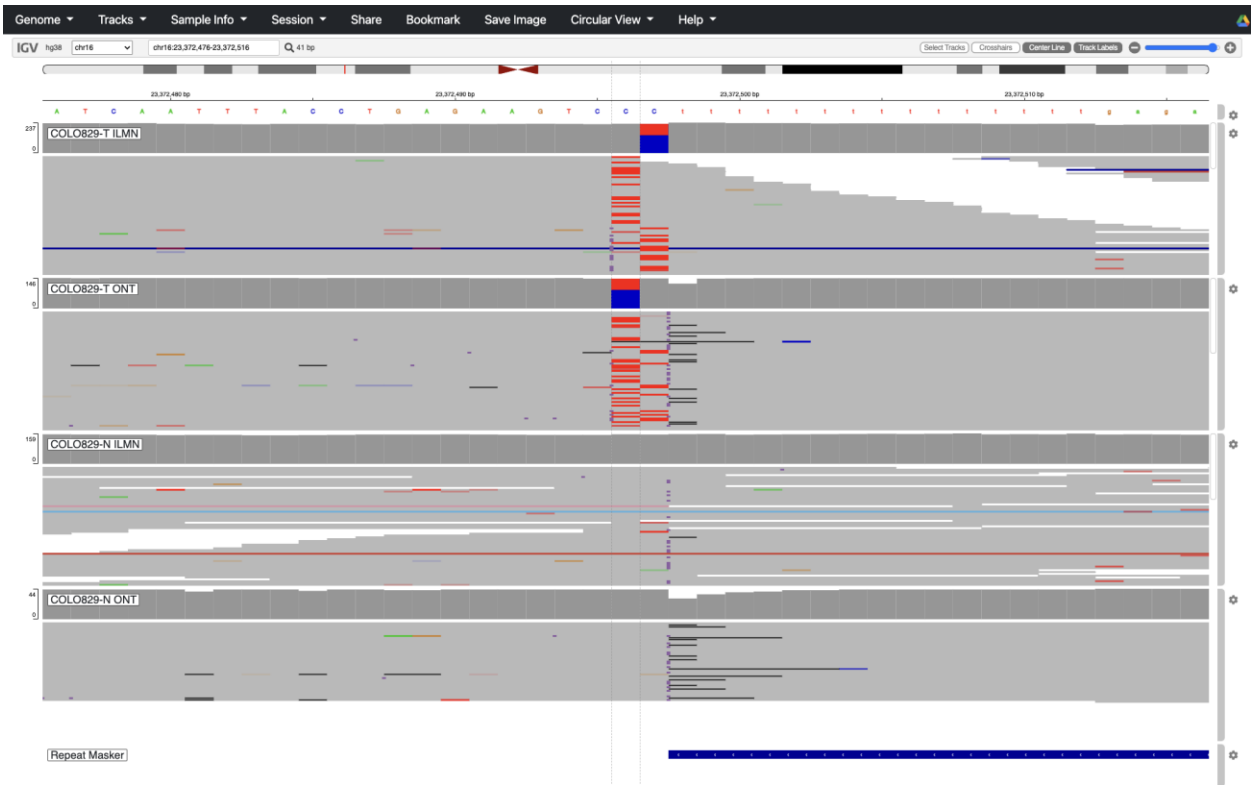

436

437 **Supplementary Figure 12: C → T somatic SNV in COLO829 at chr16:23372496 found**

438 **in the long read call set and validated by short read alignments.**

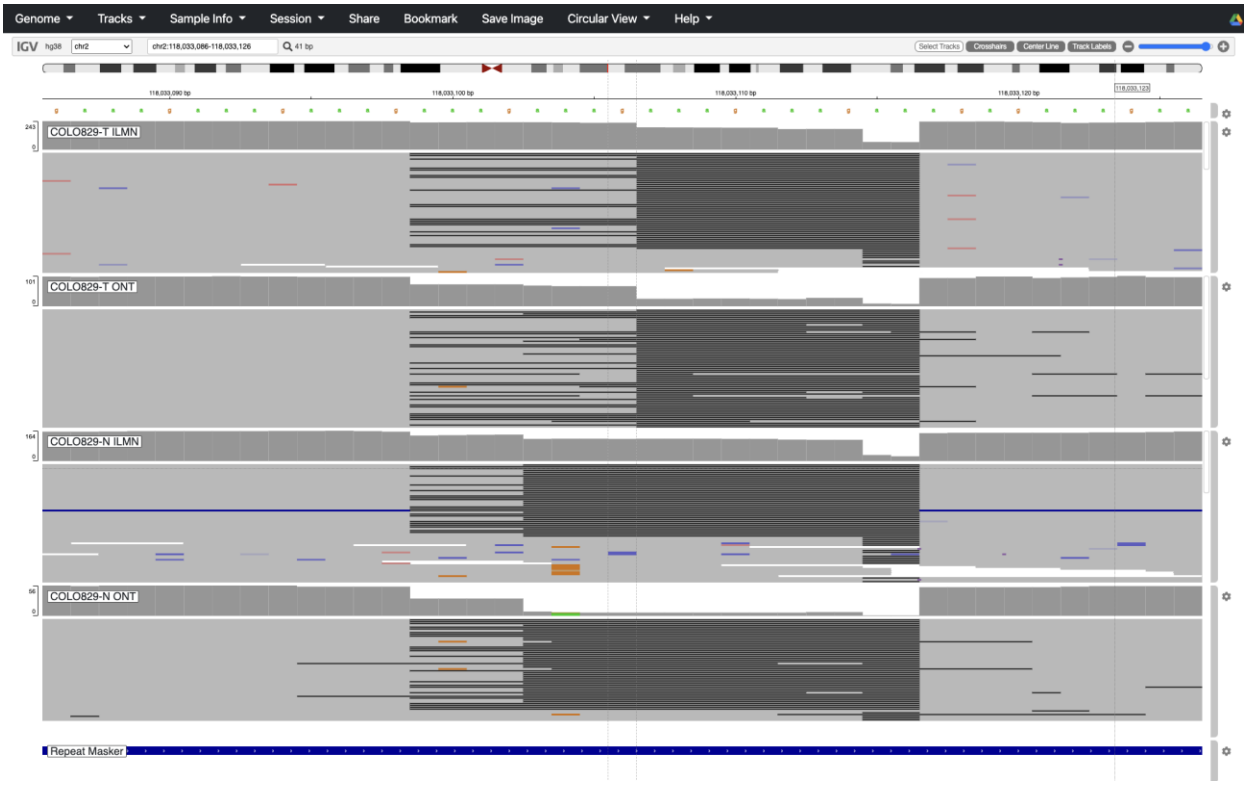

439

440 **Supplementary Figure 13: 10 bp somatic DEL in COLO829 at chr2:118033106 found in**

441 **the long read call set and validated by short read alignments.**

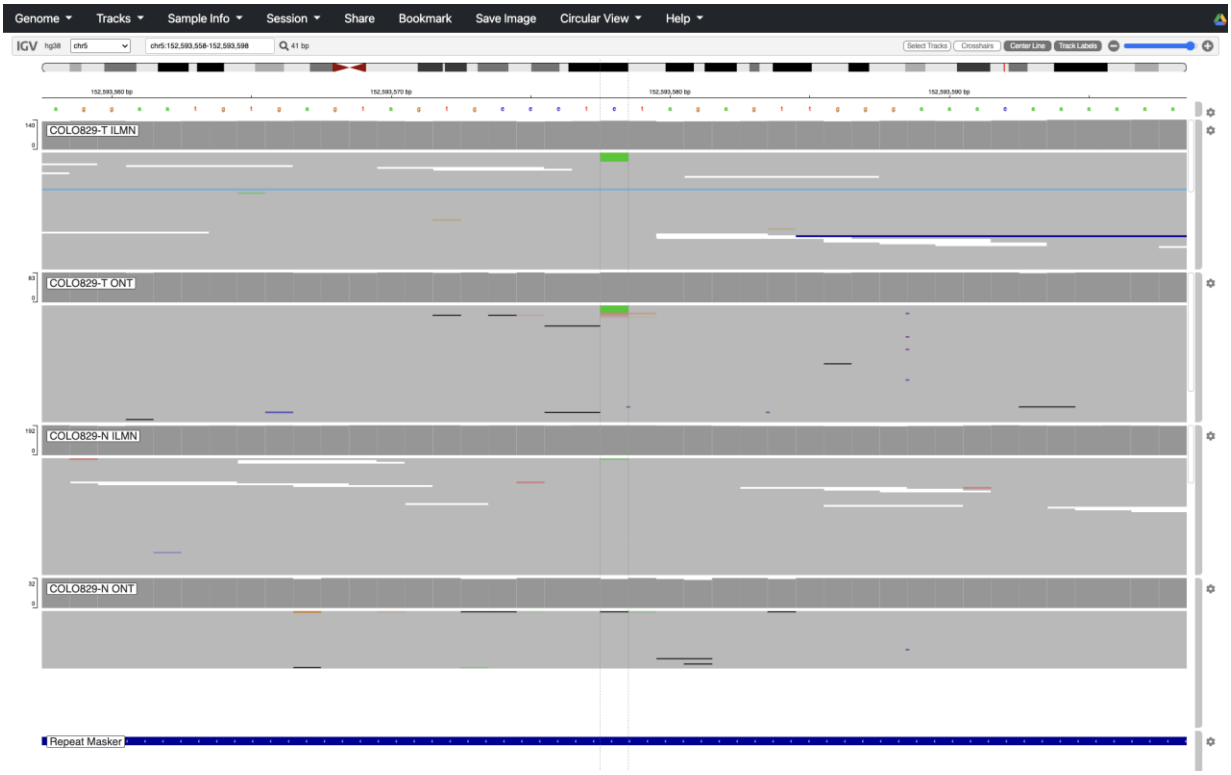

Supplementary Figure 14: C → A somatic SNV in COLO829 at chr5:152593578 found in the short read call set and validated by long read alignments.

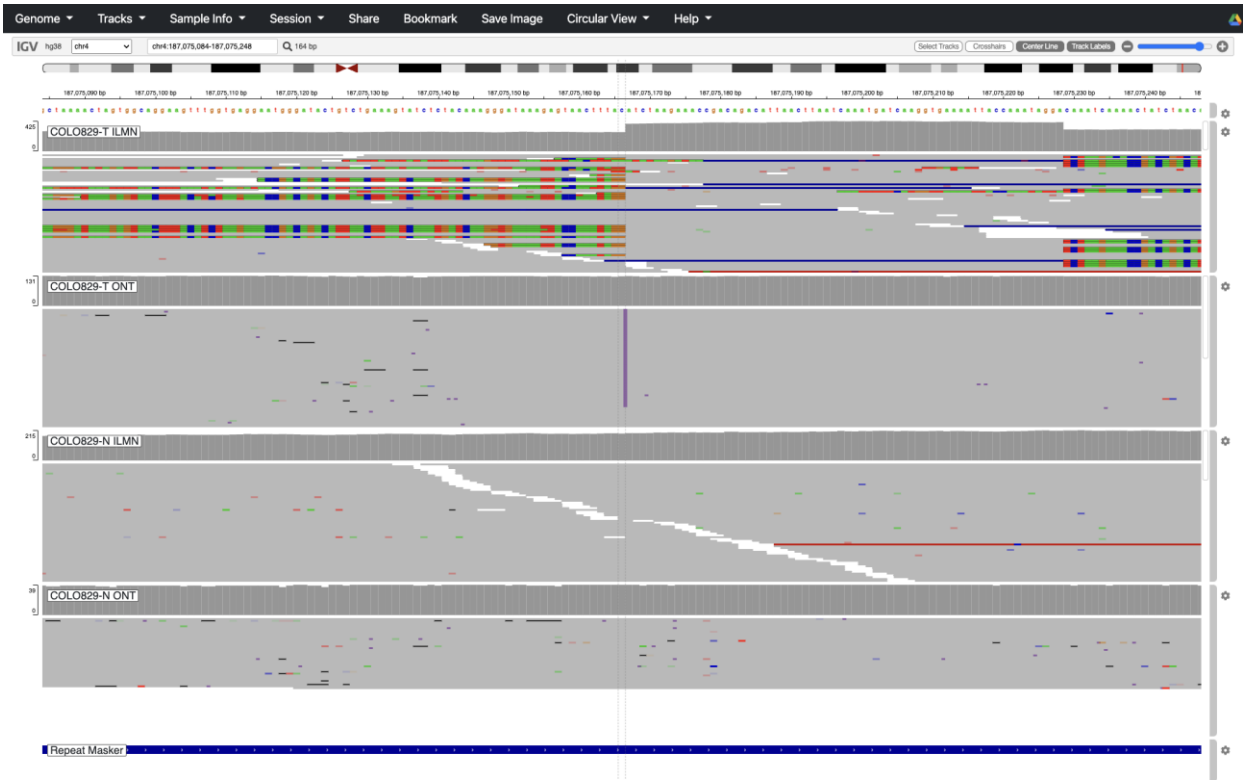

Supplementary Figure 15: 61 bp somatic INS in COLO829 at chr4:187075166 found in the short read call set and validated by long read alignments.

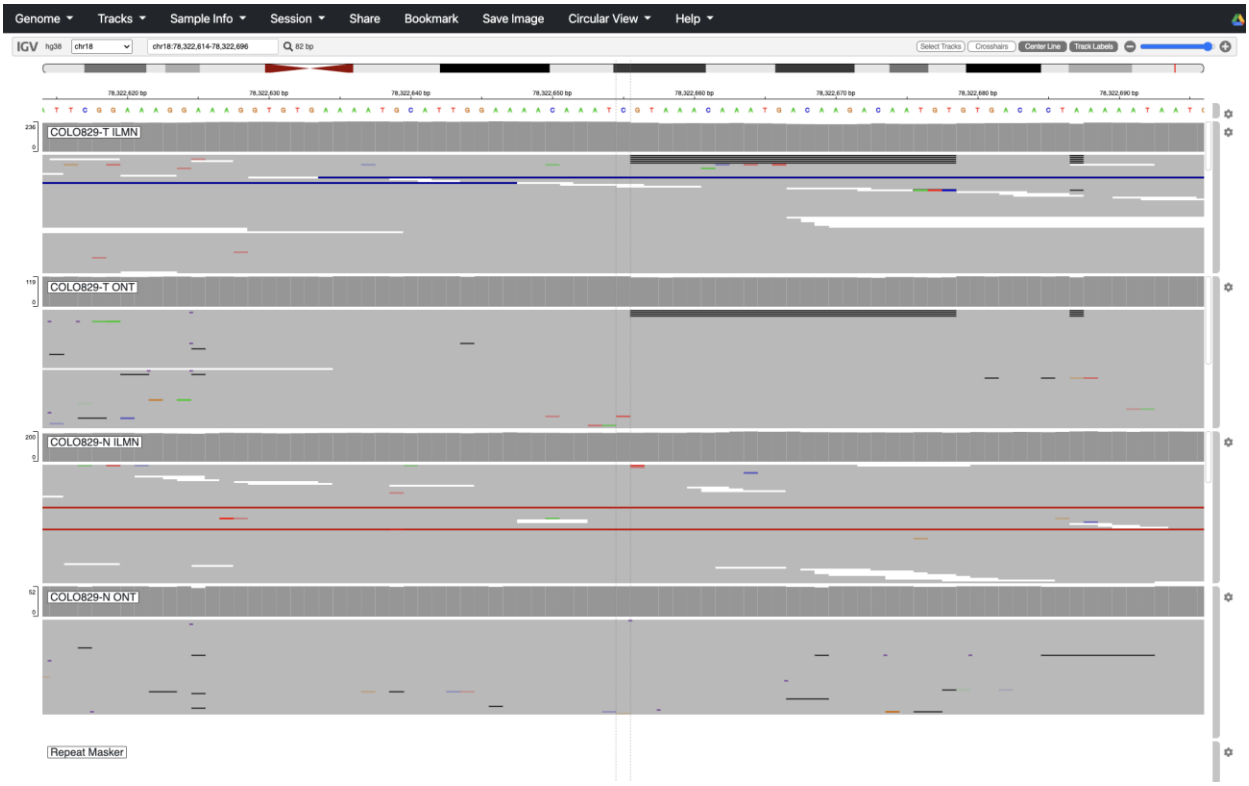

448

449 **Supplementary Figure 16: 23 bp somatic DEL in COLO829 at chr18:78322655 found in**

450 **the short read call set and validated by long read alignments.**

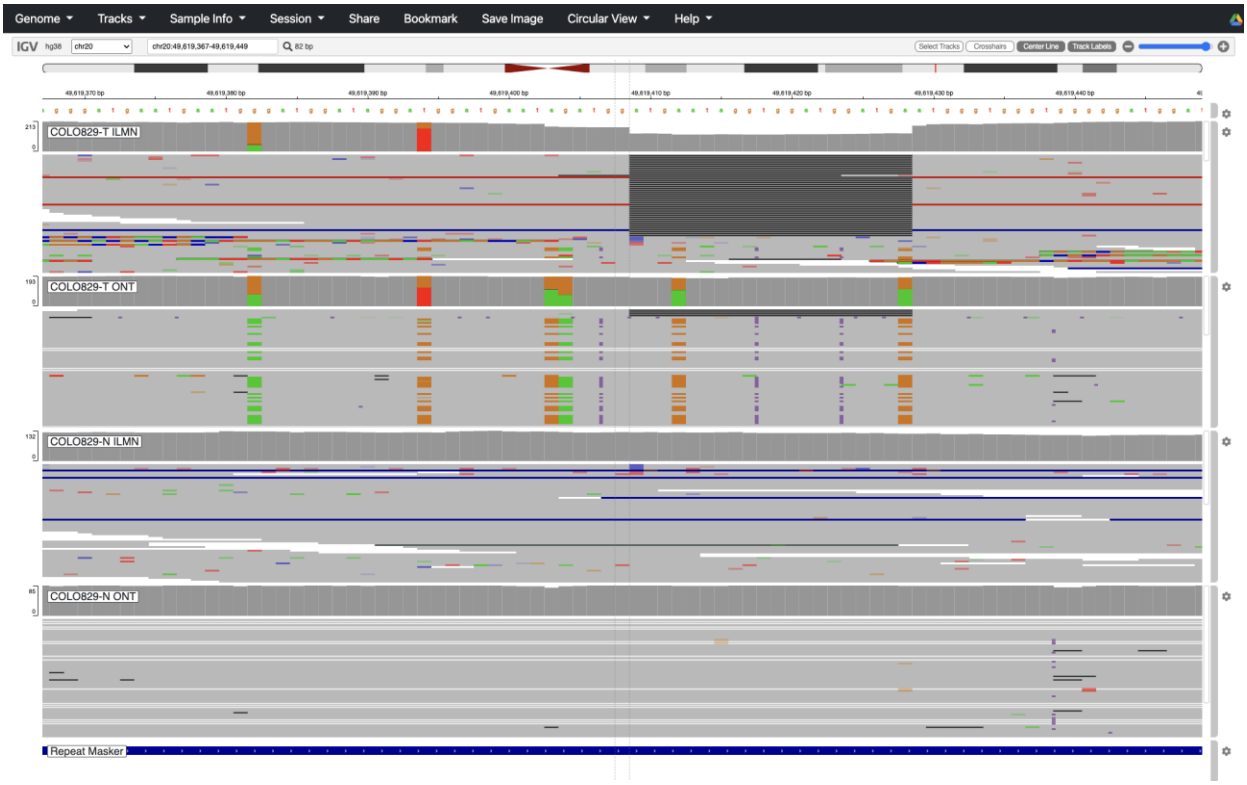

451

452 **Supplementary Figure 17: 20 bp somatic DEL in COLO829 at chr20:49619408 found in**

453 **the short read call set and validated by long read alignments.**

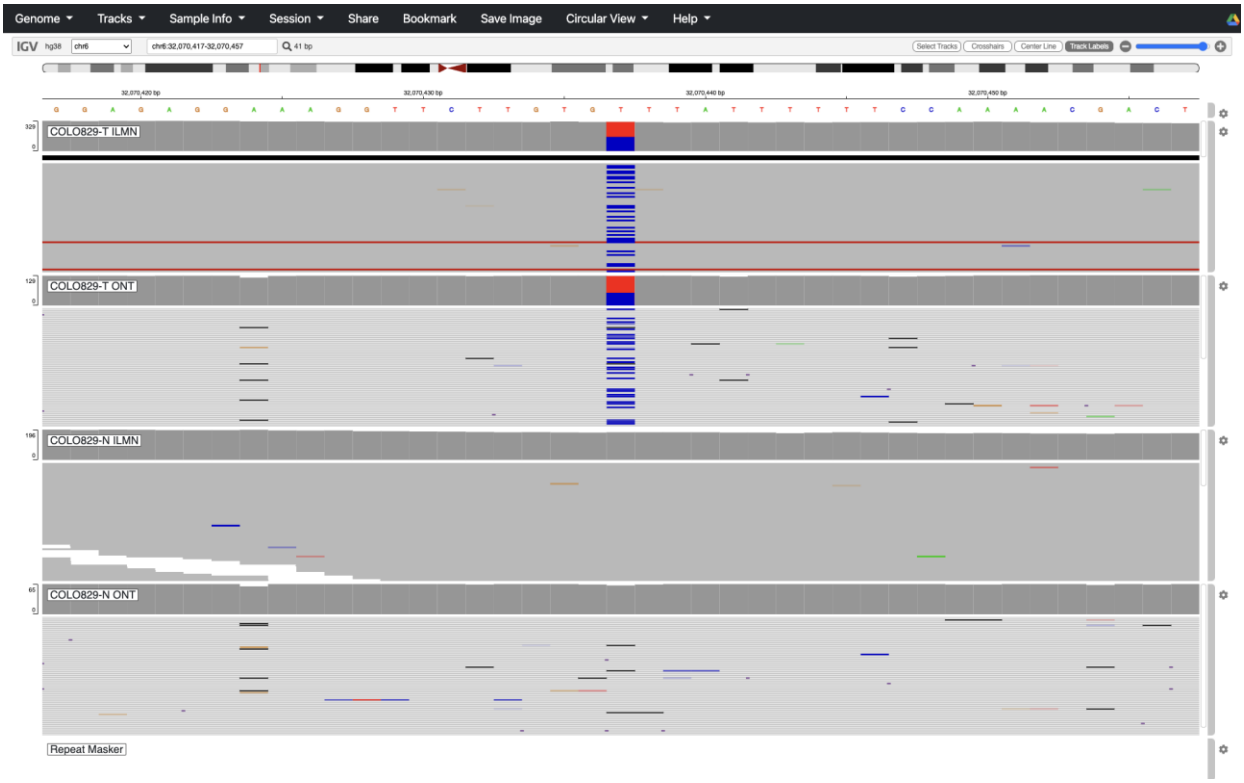

454

455 **Supplementary Figure 18: T → C putative somatic SNV in COLO829 at chr6:32070437**

456 **dropped from the previous high confidence short read truth set. Reason – only**

457 **ambiguous mapping quality zero long read alignments support ALT allele.**

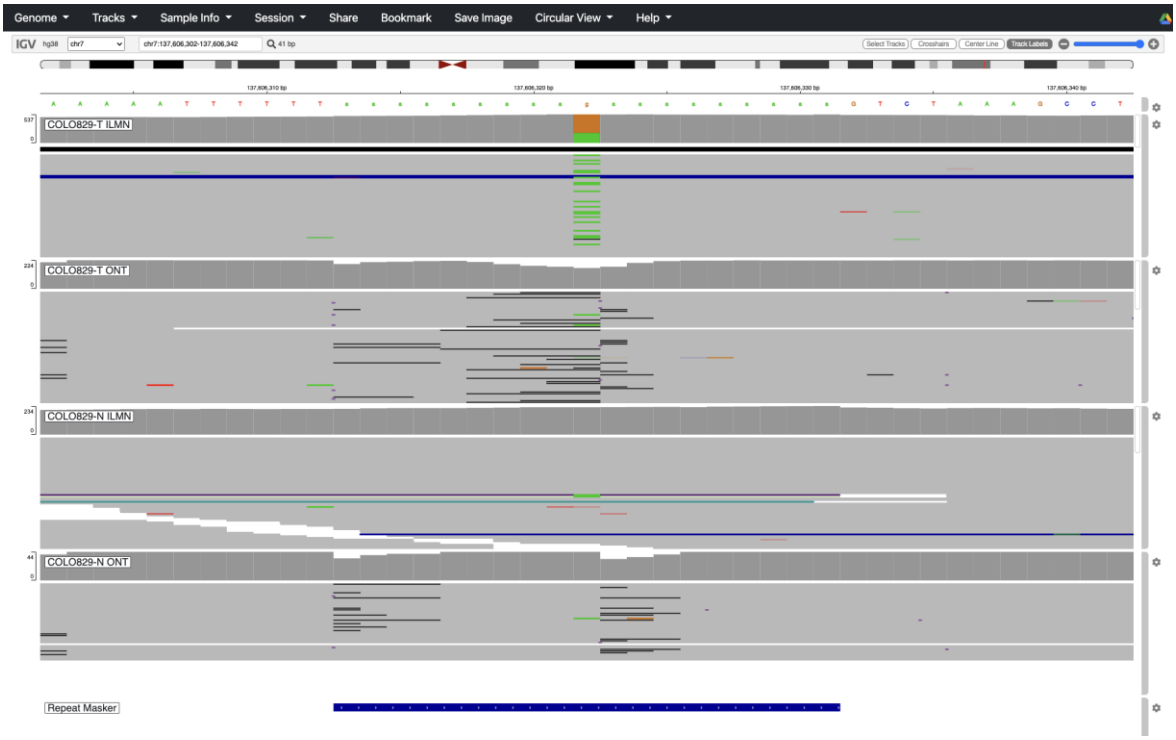

458

459 **Supplementary Figure 19: G → A putative somatic SNV in COLO829 at**

460 **chr7:137606322 dropped from the previous high confidence short read truth set.**

461 **Reason – ALT allele seen in normal sample.**

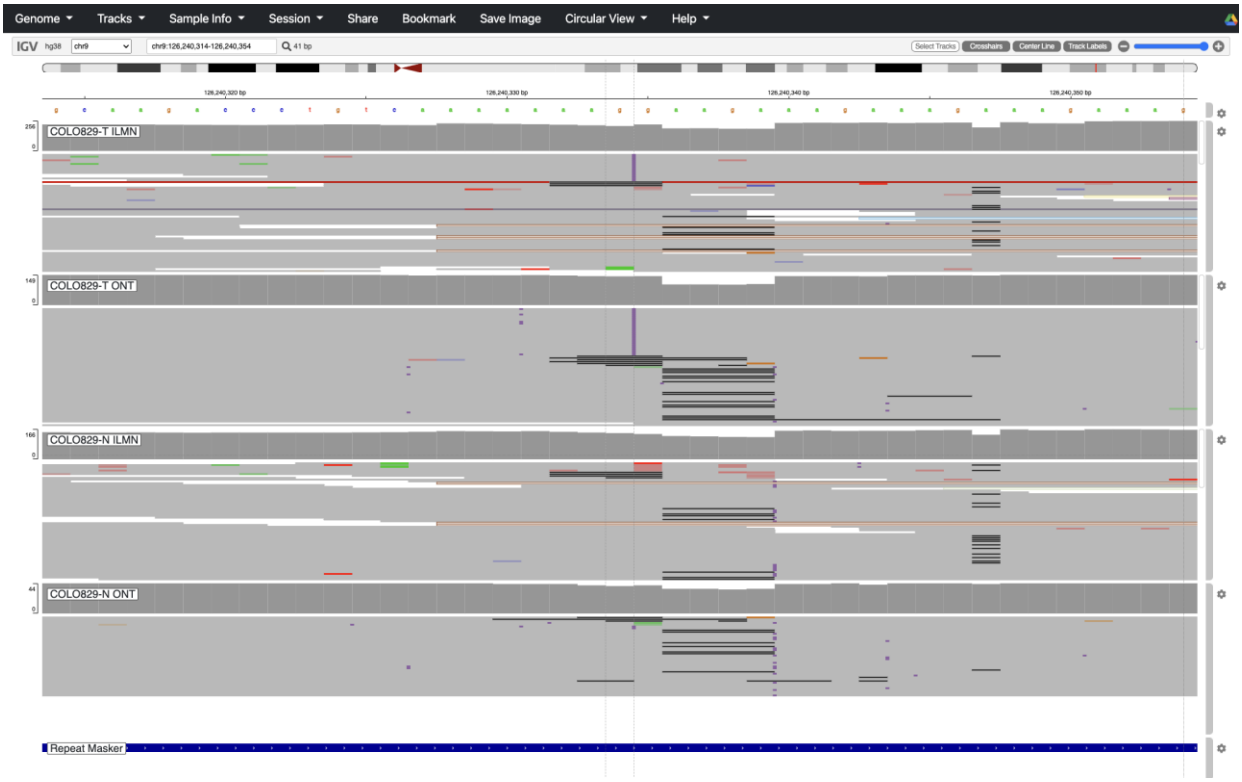

462

463 **Supplementary Figure 20: 11 bp putative somatic INS in COLO829 at chr9:126240334**

464 **dropped from the previous high confidence short read truth set. Reason – ALT allele**

465 **seen in normal sample.**

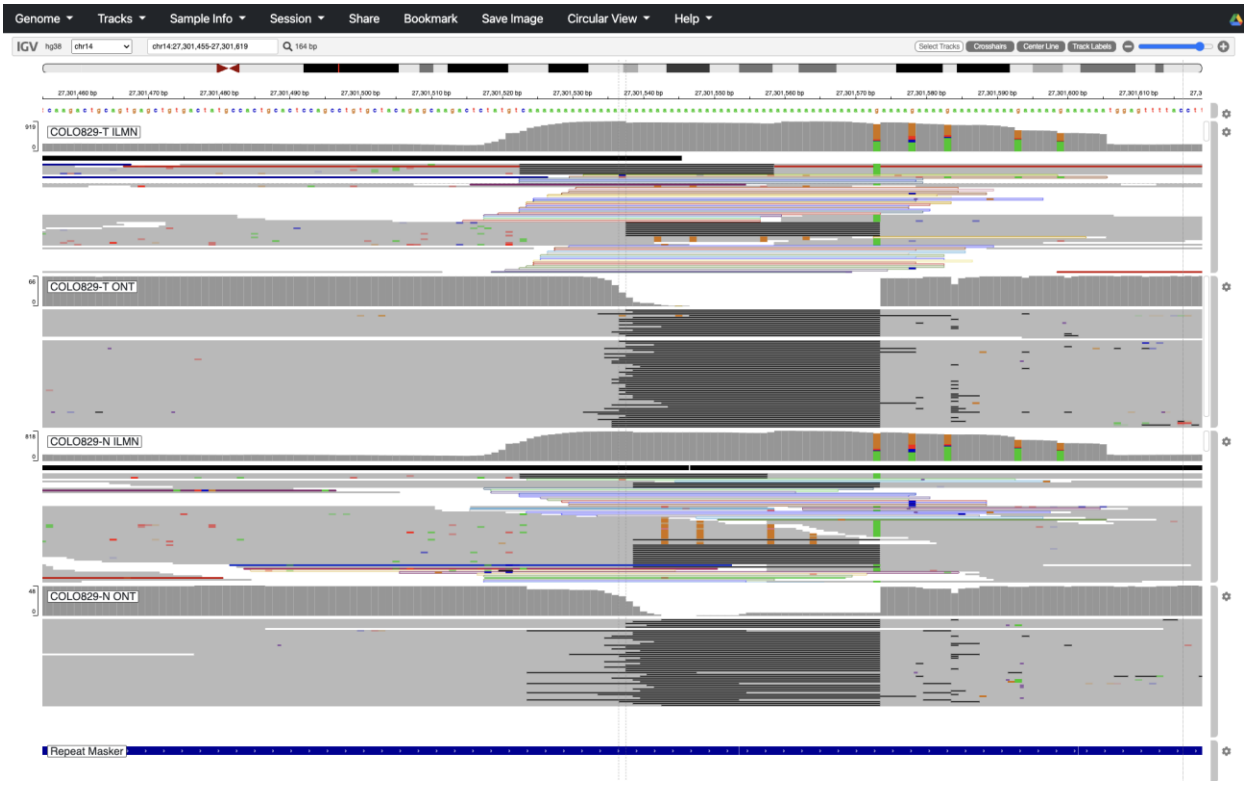

Supplementary Figure 21: 36 bp putative somatic DEL in COLO829 at chr14:27301537 dropped from the previous high confidence short read truth set. Reason – homozygous germline deletion.

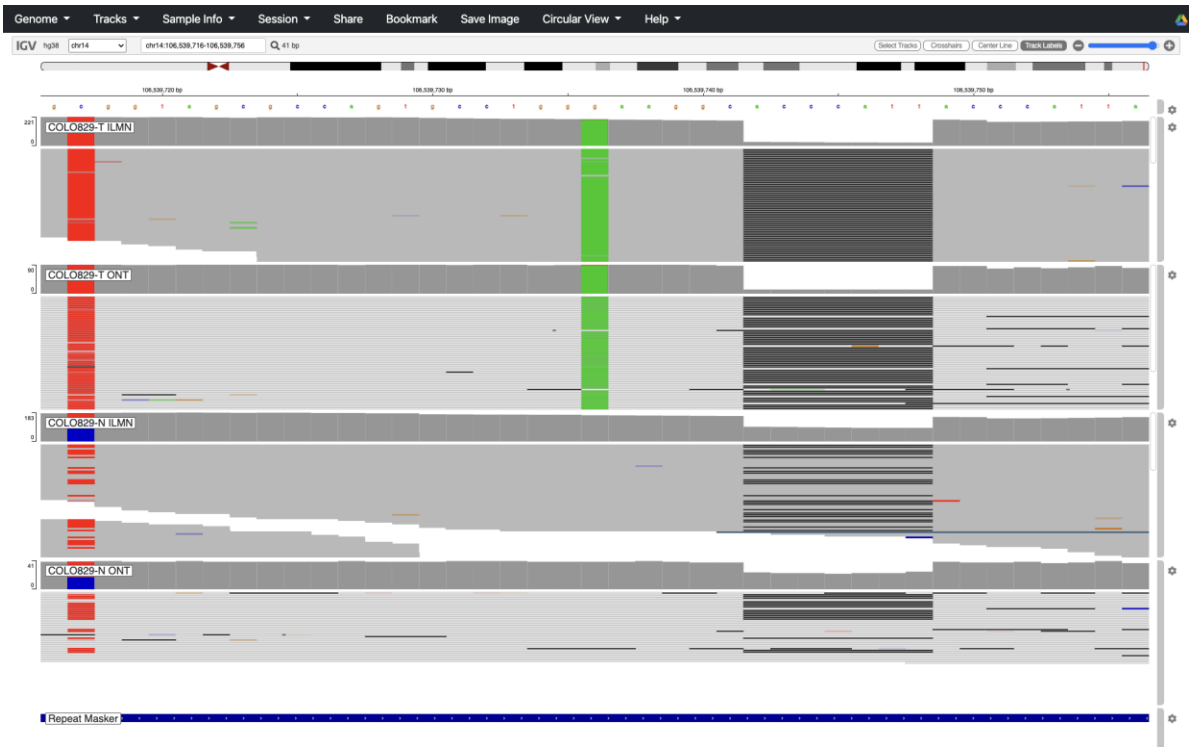

Supplementary Figure 22: G → A putative somatic SNV in COLO829 at chr14:106539736 dropped from the previous high confidence short read truth set. Reason – only ambiguous mapping quality zero long read alignments support ALT allele.

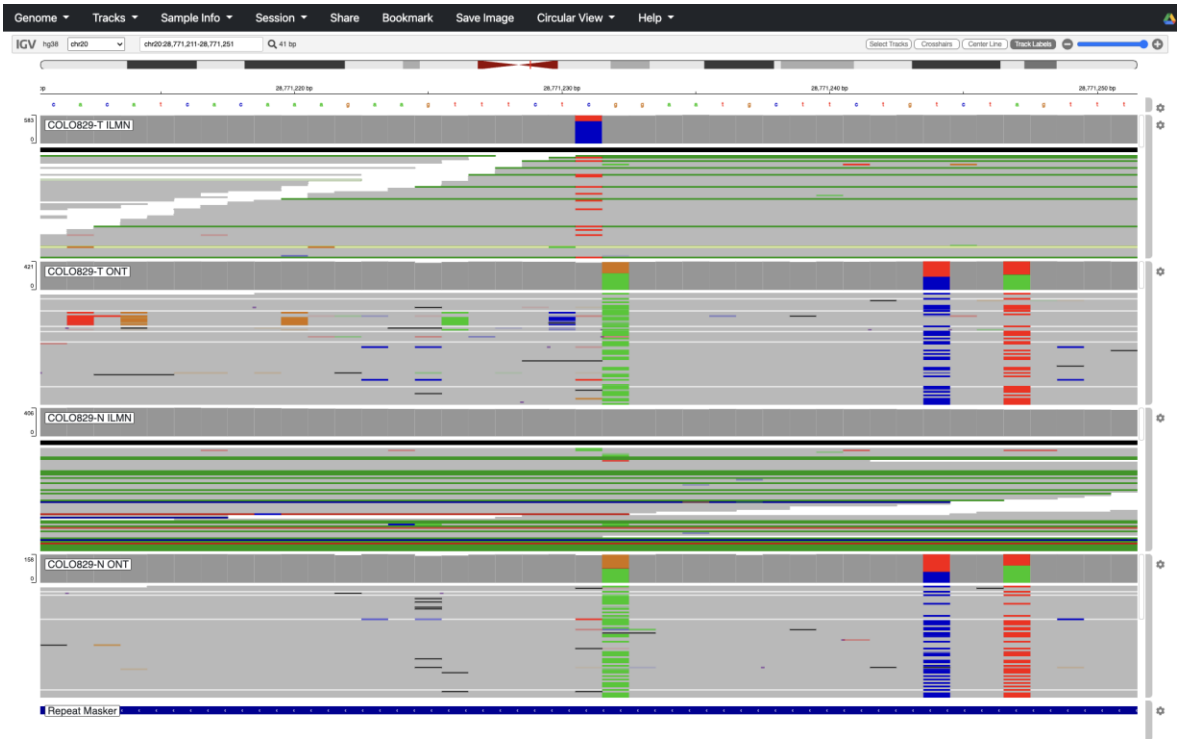

Supplementary Figure 23: C → T putative somatic SNV in COLO829 at chr20:28771231 dropped from the previous high confidence short read truth set. Reason – ALT allele seen in normal long read alignments.
